# Thraustochytrids: Evolution, Ultrastructure, Biotechnology, and Modeling

**DOI:** 10.3390/ijms252313172

**Published:** 2024-12-07

**Authors:** Aleksei G. Menzorov, Daniil A. Iukhtanov, Ludmila G. Naumenko, Aleksandr V. Bobrovskikh, Ulyana S. Zubairova, Ksenia N. Morozova, Alexey V. Doroshkov

**Affiliations:** 1 Institute of Cytology and Genetics, Siberian Branch of the Russian Academy of Sciences, 630090 Novosibirsk, Russia; menzorov@bionet.nsc.ru (A.G.M.); iukhtanov.daniil@gmail.com (D.A.I.); l.naumenko@g.nsu.ru (L.G.N.); avb@bionet.nsc.ru (A.V.B.); ulyanochka@bionet.nsc.ru (U.S.Z.); morozko@bionet.nsc.ru (K.N.M.); 2 Department of Natural Sciences, Novosibirsk State University, 630090 Novosibirsk, Russia; 3 Department of Genomics and Bioinformatics, Institute of Fundamental Biology and Biotechnology, Siberian Federal University, 660036 Krasnoyarsk, Russia

**Keywords:** marine protists, thraustochytrids, ultrastructure, phylogenetics, bioreactor

## Abstract

The thraustochytrids are a group of marine protists known for their significant ecological roles as decomposers and parasites as well as for their potential biotechnological applications, yet their evolutionary and structural diversity remains poorly understood. Our review critically examines the phylogeny of this taxa, utilizing available up-to-date knowledge and their taxonomic classifications. Additionally, advanced imaging techniques, including electron microscopy, are employed to explore the ultrastructural characteristics of these organisms, revealing key features that contribute to their adaptive capabilities in varying marine environments. The integration of this knowledge with available omics data highlights the huge biotechnological potential of thraustochytrids, particularly in producing ω-3 fatty acids and other bioactive compounds. Our review underscores the importance of a systems biology approach in understanding thraustochytrids biology and highlights the urgent need for novel, accurate omics research to unlock their full biotechnological potential. Overall, this review aims to foster a deeper appreciation of thraustochytrids by synthesizing information on their evolution, ultrastructure, and practical applications, thereby providing a foundation for future studies in microbiology and biotechnology.

## 1. Introduction

Thraustochytrids, microscopic protists belonging to the phylum Stramenopila, have received increasing attention due to their unique biological features and potential biotechnological applications. Found predominantly in marine environments, these single-celled organisms are characterized by their ability to produce large quantities of lipid-rich compounds, notably ω-3 fatty acids, which are essential for human health and are integral in various industrial applications. The significance of thraustochytrids extends beyond their nutritional benefits; they also contribute to carbon cycling in marine ecosystems and possess remarkable metabolic versatility, positioning them as a subject of interest for researchers in mycology, marine biology, and biotechnology.

Despite the early isolation and characterization of *Thraustochytrium* spp. in the 1930s [1], our understanding of their evolutionary lineage and ecological roles remains incomplete. Phylogenetic studies have provided insights into the evolutionary history of thraustochytrids, revealing their close relationship to other Stramenopiles and raising questions regarding their taxonomic classification [2,3].

Ultrastructural studies have further illuminated the unique cellular architecture of thraustochytrids, showcasing distinctive organelles such as the lipid bodies, paranuclear body, bothrosome, and ectoplasmic net. Researchers have highlighted the intricate relationships between these structures and the organisms’ metabolic pathways, sparking discussions on the evolutionary implications of such features [4,5,6]. However, fundamental questions about their physiological adaptations and ecological functions continue to be the subject of ongoing research and debate.

In the field of biotechnology, there is growing interest in harnessing the capabilities of thraustochytrids for the sustainable production of high-value products. A recent review emphasized the need for modern molecular technologies, omics data, and visualization technologies to be applied to study their peculiarities in detail [7]. Therefore, a comprehensive review that synthesizes the existing knowledge on the phylogeny, ultrastructure, and biotechnological applications of thraustochytrids is especially relevant.

The primary aim of this article is to compile and critically analyze the current research on thraustochytrids, highlighting their evolutionary background, structural characteristics, and biotechnological significance. By providing a thorough overview of the state of the art, this review seeks to bridge gaps in the current understanding of these versatile microorganisms. Ultimately, we conclude that thraustochytrids represent a valuable resource not only for advancing scientific knowledge but also for facilitating innovations in biotechnology that could lead to sustainable solutions for global challenges.

## 2. Phylogeny

The first descriptions of species within the genus *Labyrinthula* appeared in 1867 [8]. Initially, these species were classified as Myxomycetes, while *Thraustochytrium proliferum* sp., described by Sparrow in 1936 along with the new genus *Thraustochytrium*, was placed in the Phycomycetes [1]. For a long time, labyrinthulids and thraustochytrids were classified within the Oomycetes. It was only in 1975 that these two taxa were combined into a single class [9]. Subsequent morphological studies and phylogenetic analyses further refined the classification, establishing *Labyrinthulea* within the Heteroconta as a distinct group separate from the Oomycetes [10,11,12,13]. Currently, the class Labyrinthulea is included in the paraphyletic group Bigyra, which comprises the clades Sagenista (containing Labyrinthulea) and Opalozoa [14,15].

The repeated revision of the classification of Labyrinthulea has been largely due to the limitations of traditional taxonomic approaches. In the pre-genomic era, the taxonomy of the class Labyrinthulea relied on morphological and life cycle characteristics, including the presence or absence of specific developmental stages and the ability to utilize various substrates. However, key morphological and life cycle traits in protists, which are essential for classification, are often difficult to observe under laboratory conditions. For example, the morphology of thraustochytrids is highly influenced by environmental factors, which can significantly affect conclusions about their phylogenetic relationships when based solely on morphological criteria [16,17].

While the position of Labyrinthulea on the tree of life is now generally accepted, the classification of taxa within Labyrinthulea has remained a challenging issue. One of the first studies to question the traditional morphology-based taxonomy was Honda’s 1999 work [18], which identified two major phylogenetic groups within the class: the labyrinthulid phylogeny group and the thraustochytrid phylogeny group. The study also demonstrated that species previously classified within the same genus may belong to different phylogenetic groups, highlighting the need for a comprehensive taxonomic revision. The reclassification effort initiated by Honda in 1999 [18] is still ongoing. Molecular phylogenetic studies aiming to clarify the taxonomic positions of newly discovered species frequently reveal heterogeneity within existing genera, with species from different genera appearing as closest relatives within the same clade [16,17,19,20]. Phylogenies incorporating biochemical markers are generally more robust, and some researchers suggest using fatty acid metabolism profiles as additional markers to distinguish between the phylogenetic groups within a class [21].

Currently, the division of the class Labyrinthulea into two primary orders, Labyrinthulida and Thraustochytrida, is well established. Early analyses of 18S rRNA identified order Aplanochytrida as a monophyletic group within Labyrinthulida, thereby completing the labyrinthulid phylogeny group [22]. Presently, only one genus, *Aplanochytrium*, is recognized in order Aplanochytrida [23]. Additionally, Labyrinthulida includes two stable genera: *Labyrinthula* sp. and *Stellarchytrium* sp. [24]. Three more orders lie outside the labyrinthulids and thraustochytrids phylogeny groups. The first, order *Amphitremida* [25], comprises four genera: *Amphitrema*, *Archerella*, *Diplophrys*, and *Paramphitrema*. The second, known as *Amphifilida* [19], includes three genera: *Amphifila*, *Fibrophrys*, and *Sorodiplophrys*. The third order, Oblongichytrida, contains the single genus *Oblongichytrium* [24,26]. Within order Thraustochytrida, most researchers recognize nine main genera: *Thraustochytrium*, *Japonochytrium*, *Schizochytrium*, *Ulkenia*, *Aurantiochytrium*, *Sicyoidochytrium*, *Parietichytrium*, *Botryochytrium*, and *Monorhizochytrium*. Additionally, two closely related genera, *Oblongichytrium* and *Althornia*, could be included or excluded from this order [6,21]. Furthermore, in 2018, Dellero et al. proposed a new genus within *Thraustochytrida*, named *Hondea* gen. nov [27].

The relative positions of these groups vary depending on the data used in phylogenetic reconstruction. Certain genera have been reassigned to different families based on new phylogenetic evidence [25]. For instance, several species within the genus *Thraustochytrium* are closely related to species in other genera.

Currently, 18S ribosomal RNA (rRNA) sequencing is a standard method for characterizing new isolates. To assess the current phylogenetic relationships among Labyrinthulea samples, we accessed the GenBank database [28], which, as of 16 July 2024, contained 2914 18S rRNA sequences representing Labyrinthulea species. The sequences for all Labyrinthulea species were retrieved using a BLAST search within the Bigyra taxon in the NCBI database. The detailed protocol is provided in Appendix A.1, and the sequence IDs are listed in Supplementary Table S1.

**Figure 1:**
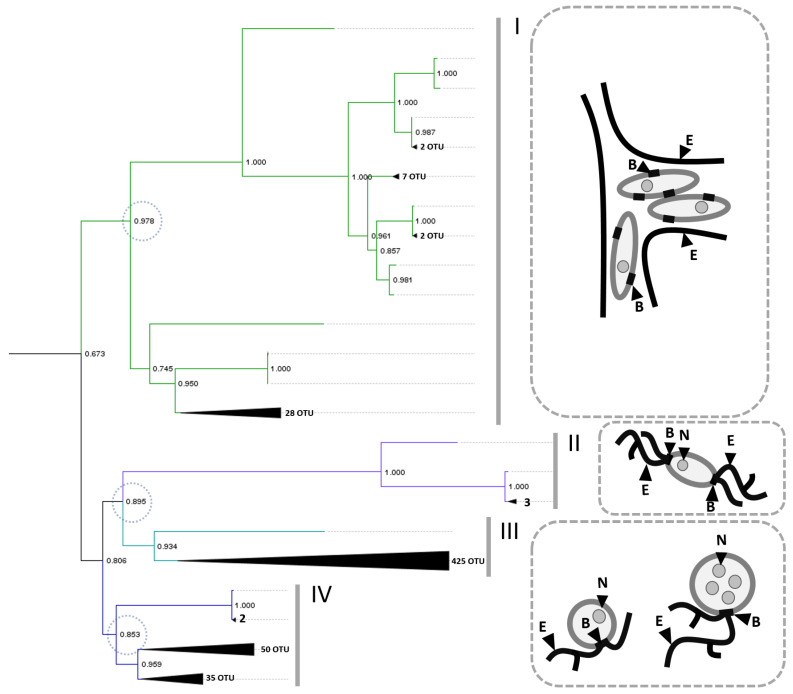
Phylogenetic tree of the Labyrinthulea class. The tree was constructed using available 18S rRNA sequences from the NCBI Gene Database (https://www.ncbi.nlm.nih.gov/gene, accessed on 15 April 2024) by the maximum likelihood method. This tree is divided into four clades, representing the main families: The first clade (I) consists of species from the order Labyrinthulida (50 sequences). The second clade (II) includes species from the order Amphifilida: *Sorodiplophrys stercorea* sp. (four sequences) and *Amphifilidae* sp. (one sequence). In the third clade (III), order Thraustochyrida, 132 of the 426 sequences correspond to the *Aurantiochytrium* spp., 118 belong to the *Thraustochytrium* spp., 72 are designated as *Thraustochytriidae* spp., 33 sequences to the *Ulkenia* spp., 30 sequences to the *Schizochytrium* spp., 11 sequences to *Thraustochytriaceae* spp., and several sequences correspond to other species. The fourth clade (IV) is represented by orders Oblongichytrida and Aplanochytrida. In total, 54 of the 88 sequences belong to the *Oblongichytrium* spp., and 25 sequences belong to the *Aplanochytrium* spp. E—ectoplasmic network, B—bothrosome, N—nucleus.

Based on these publicly available data, we reconstructed a phylogenetic tree for all isolates with a sufficiently long 18S rRNA sequence. Figure 1 displays the basal topology of the reconstructed phylogenetic tree, highlighting four distinct clades containing representatives from different families (marked with Roman numerals). The statistical support for the basal nodes of these clades exceeds 0.85, indicating their stability.

Clade I is represented exclusively by specimens from the order Labyrinthulida and contains 50 sequences (for a list of sequences and their species assignments, see Supplementary Table S1 and Figure S1). This family is characterized by a unique structure of cellular aggregates: the cells possess multiple bothrosomes and are situated within a large membrane structure formed by the ectoplasmic reticulum, along which the cells can move [29]. On the one hand, this feature complicates the biotechnological applicability of these species; on the other hand, the presence of an additional compartment sometimes enables the synthesis of substances that may be toxic to the producer cell.

Another monophyletic group is represented by three daughter clades (II–IV). A distinctive feature of this group is the formation of the ectoplasmic network. It does not surround the cells but rather forms a separate structure of branching threads (see Figure 1).

Clade II contains four sequences belonging to the species *Sorodiplophrys stercorea* sp. of the order Amphifilida and one sequence identified as *Amphifilidae* sp. (for the list of sequences and their species assignments, see Supplementary Table S1 and Figure S2). Representatives of this family form ectoplasmic networks outside the cell as filamentous structures resembling microscopic mycelium. They possess two polar bothrosomes, which serve as attachment points for the ectoplasmic networks [30].

Clade III, a sister clade to II, contains all samples from the order Thraustochyrida, totaling 426 sequences. Among these sequences, 118 were assigned to the *Thraustochytrium* spp., 72 to the *Thraustochytriidae* spp., 30 to the *Schizochytrium* spp., 11 to the *Thraustochytriaceae* spp., 5 to the *Parietichytrium* spp., 5 to the *Botryochytrium* spp., 4 to the *Sicyoidochytrium* spp., 3 to the *Monorhizochytrium globosum* spp., 1 to the *Japonochytrium* spp., 1 to the *Labyrinthulochytrium arktikum* spp., 1 to the *Phycophthorum isakeiti* spp., 1 to *Hondaea fermentalgiana*, 33 to the *Ulkenia* spp., and 132 to the *Aurantiochytrium* spp. (for a list of sequences and their species assignments, see Supplementary Table S1 and Figure S3). These are the most common Labyrinthulea isolates in laboratory culture, likely due to their widespread distribution and ease of cultivation [20]. Members of this clade possess a single bothrosome that serves as the formation point for the ectoplasmic reticulum, and their life cycle includes large vegetative cells with multiple nuclei [31].

Clade IV contains 88 sequences, including those from the orders Oblongichytrida (54 sequences) and Aplanochytrida (25 sequences). This clade comprises species that were traditionally assigned to the *Oblongichytrium* but still retain trivial names from other genera (e.g., *Schizochytrium minutum* and *Thraustochytrium multirudimentale*). Additionally, it includes one species assigned to the order Labyrinthulida (*Stellarchytrium dubum*), a species from the class Bicosoecida rather than Labyrinthulea (*Adriamonas peritocrescens*), and four sequences designated as *Thraustochytriidae* spp. (for a list of sequence IDs and their species assignments, see Supplementary Table S1 and Figure S4). This composition may suggest either an incomplete resolution of the phylogeny within Clade IV or a potential misassignment of species based on morphological characteristics. These species also exhibit a vegetative cell morphology with a single bothrosome and an attachment site for the ectoplasmic reticulum [31].

Thus, the currently available isolates with known 18S rRNA sequences are clustered into two large groups. Clade I corresponds to the order Labyrinthulida, while its sister clade represents order Thraustochytrida, which aligns with Honda’s original classification (1999) [18] and reflects significant morphological differences among these organisms. However, some discrepancies exist compared to the previous literature [23]; for instance, orders Oblongichytrida and Aplanochytrida are grouped, even though Aplanochytrida has traditionally been considered a sister group to the order Labyrinthulida. Moreover, they are positioned closer to the Thraustochytrida phylogenetic group than to Labyrinthulida.

In our classification, order Thraustochytrida forms three subgroups that generally align with the morphological data. However, when examining more recent common ancestors (e.g., at the genus level), the stability of the nodes decreases. This supports the reliability of using 18S rRNA primarily for basal classification within Labyrinthulea; for resolving shorter evolutionary distances, additional gene sequences are necessary. In the future, we propose using the division into four groups:

I    Labyrinthulida;
II  Amphifilidae;
III Thraustochytrida, including the *Thraustochytrium* spp., *Schizochytrium* spp., *Parietichytrium* spp., *Botryochytrium* spp., *Sicyoidochytrium* spp., *Monorhizochytrium* spp., *Japonochytrium* spp., *Labyrinthulochytrium* spp., *Phycophthorum* sp., *Hondaea* spp., *Ulkenia* spp., and *Aurantiochytrium* spp.;
IV containing the orders Oblongichytrida and Aplanochytrida.

## 3. Life Cycle and Ultrastructure

**Figure 2:**
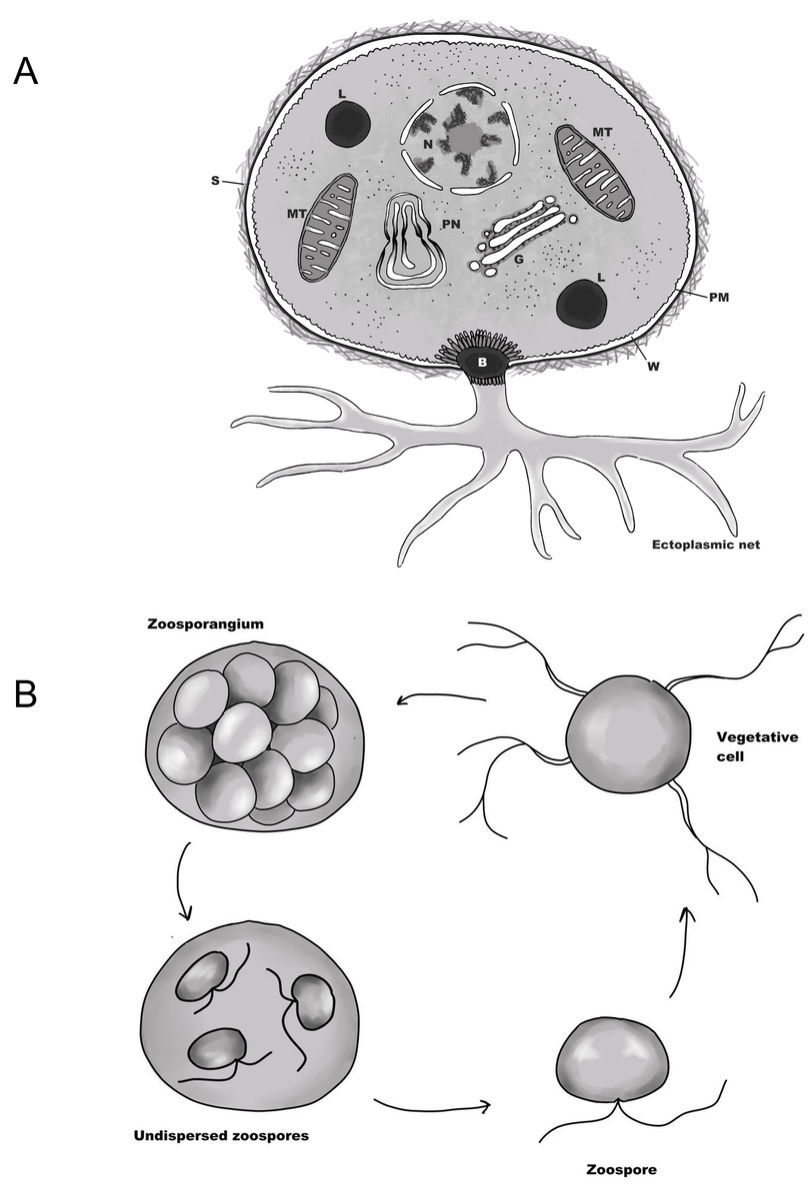
Scheme of the ultrastructural organization of the cell (**A**) and the minimal life cycle (**B**) of thraustochytrids. Cell structure (**A**): S—scales; W—cell wall; PM—plasma membrane; MT—mitochondria; L—lipid droplets; N—nucleus; G—Golgi complex; PN—paranuclear body; B—bothrosome. Life cycle (**B**): the typical cell development and replication phase stages of the life cycle of thraustochytrids.

To date, the ultrastructure of numerous genera within class Labyrinthulea has been examined using electron microscopy. In this review, we outline the main ultrastructural Labyrinthulea characteristics commonly utilized in biotechnological and taxonomic assessments. Detailed descriptions of Labyrinthulea ultrastructure are provided in Figure 2 and Supplementary Table S2, and the method for article selection is described in Appendix A.2.

### 3.1. Life Cycle

In the genera of the Labyrinthulea, life cycles typically share several common cell development pathways. In thraustochytrids, there are usually three main stages: vegetative cells, a multinucleate stage (zoosporangium) or aplanospores, followed by cytoplasmic cleavage, which results in the formation of motile zoospores [21] (see Figure 2). In Labyrinthulids, except for the genus *Aplanochytrium*, nonmotile aplanospores are included in the life cycle [26]. Notably, some genera, such as *Botryochytrium*, *Parietichytrium*, *Sicyoidochytrium*, and *Ulkenia*, exhibit more complex life cycles that include free-living amoeboid cells [26]. Additionally, *Aurantiochytrium acetophilum* sp. demonstrates a variety of developmental pathways, including for sexual reproduction [32].

Like most eukaryotes, Labyrinthulea possesses essential organelles, including a nucleus, mitochondria, a Golgi complex, lipid bodies, and rough and smooth endoplasmic reticula. In addition to these classic cellular components, some Labyrinthulea species have paranuclear bodies—regions of convoluted, smooth endoplasmic reticulum closely associated with the nuclear envelope, enclosing ribosome-free cytoplasm [33]. In *Fibrophrys columna* sp. [34] and *Diplophrys mutabilis* sp. [35], unidentified cytoplasmic membranes are found. These undefined structures are ribosome-free and connected with lipid bodies and the outer mitochondrial membrane via the endoplasmic reticulum. Additionally, Labyrinthulea cells have extracellular structures such as ectoplasmic nets and scales, which are unique to this class of protists [26].

The outer ectoplasmic net, likely responsible for nutrient intake, enzyme secretion, absorption, and food sensing [36], forms during the vegetative cell stage. The zoosporangium, the largest cell type, measures from 6 to 50 µm, while zoospores—the smallest stage, ranging in height from 1.5 to 7.0 µm and in length from 3.0 to 8.0 µm—possess specialized flagella for movement and colonization. Both stages have been observed in seawater and cultures of pine pollen grains from the genera *Thraustochytrium*, *Schizochytrium*, *Aplanochytrium*, and *Ulkenia* [37].

Below, we summarize the key features, including the noncellulosic cell wall, ectoplasmic net, paranuclear bodies, and the characterization of lipid bodies, that have been highlighted by Loris Fossier Marchan et al. [21] and Yokoyama et al. [38].

### 3.2. Lipid Bodies

Organisms in the *Labyrinthulea* clade are active producers of fatty acids with lipid bodies of various sizes and shapes within their cells. In *Schizochytrium limacinum* sp. SR21 [39] and *Aurantiochytrium mangrovei* sp. MP2 [5], fine structures with light- and dark-staining bands have been observed within the lipid bodies across various stages of cell development. These bands may correspond to the ratio of saturated and monounsaturated fatty acids to highly unsaturated fatty acids, as osmium tetroxide can bind to the double bonds in unsaturated fatty acid chains [5,39].

Additionally, in *Aurantiochytrium mangrovei* sp. MP2 [5], lipid bodies were observed to divide into two layers with differing opacities during orbital shaking, potentially indicating differences in fatty acid saturation, since osmium tetroxide preferentially binds to double bonds in unsaturated fatty acids [40]. The variations in lipid body opacity may reflect differences in morphology and lipid composition during fermentation, with separation into two distinct layers achievable only after centrifugation [5].

Furthermore, ultrastructural analysis can be combined with other lipid screening methods. For instance, using electron microscopy alongside confocal microscopy and FACS, combined with two different lipophilic fluorescent dyes (BODIPY 505/515 and Nile Red), researchers documented the appearance, development, and degeneration of lipid bodies during the midexponential, early, and late stationary phases of *Aurantiochytrium* sp. KRS101 culture growth, respectively [41]. Additionally, changes in lipid accumulation were systematically studied through a combination of transmission electron microscopy and Nile Red staining in *Schizochytrium mangrovei* sp. PQ6 cell cultures [42]. In this study, transmission electron microscopy revealed differences in the synthesis and deposition of lipid bodies in cells grown under different fermentation conditions, batch or fed-batch.

The formation of lipid bodies was also observed in *Schizochytrium limacinum* sp. SR21 [43] and *Thraustochytrium aureum* sp. (ATCC 34304) [44], where lipid bodies were surrounded by the endoplasmic reticulum at all life cycle stages. In *Thraustochytrium* sp. (ATCC 26185), unusual, branched, hollow membrane-like structures with a crystalline appearance were associated with lipid formation [4].

### 3.3. Ectoplasmic Nets

In addition to the intracellular structures mentioned earlier, nearly all genera of Labyrinthulea possess an ectoplasmic net, which is an extracellular extension of the cell formed by the plasma membrane. This net originates from electron-dense stacks of the endoplasmic reticulum, commonly known as the sagenetosome or bothrosome. Most thraustochytrids have a single bothrosome, whereas Labyrinthulids typically possess several bothrosomes [38].

The process of bothrosome formation remains incompletely understood. Recent studies on *Schizochytrium aggregatum* sp. have shown that bothrosome formation begins at the anterior–ventral pole of the cell [45]. In addition, in *Ulkenia visurgensis* sp. zoosporangia, parallel cisternae of rough endoplasmic reticulum extend from the paranuclear body and transition into smooth endoplasmic reticulum as they approach the bothrosome [46]. A similar role of the perinuclear continuum in bothrosome formation has been observed in *Labyrinthula algeriensis* sp. and *Labyrinthula minuta* sp. [29], along with associations of the endoplasmic reticulum cisternae [47].

Several studies have shed light on the function of ectoplasmic nets. In some organisms, these nets facilitate the parasitic invasion of diatom cells [48], a function also observed in *Schizochytrium aggregatum* sp. [36]. These findings support the hypothesis that ectoplasmic nets serve a feeding function, providing new insights into their role. Specifically, the attachment site of the ectoplasmic net to a food source is surrounded by fibrous materials, and vesicles within the net may be released into the food source space. The ultrastructural characteristics of the ectoplasmic net in *Schizochytrium aggregatum* and related organisms were comprehensively summarized by Iwata et al. [36] (Table 2). It is also notable that *Thraustochytrium* sp. (ATCC 26185) lacks an ectoplasmic net [4].

### 3.4. Scales

Another distinctive feature of Labyrinthulea is their noncellulosic cell wall, which includes scales that are considered components of the cell wall structure. This cell wall is composed of sulfated polysaccharides, containing galactose and xylose in thraustochytrids and fucose in Aplanochytrids and Labyrinthulids [21,26]. Scales appear at various stages of the life cycle, though their presence is not consistent across all members of a family [49]. For instance, an ultrastructural study observed scales in the zoospore stage of *Thraustochytrium* spp. and *Schizochytrium* spp. [50]. However, another study on *Schizochytrium aggregatum* sp. found that zoospores lacked scales, with scale formation beginning in newly encysted zoospores that already possessed elements of the outer cell wall [51]. Similarly, it has been suggested that scales on the parental cells of *Diplophrys mutabilis* disappear during cell division [35].

Previous studies have documented the structural elements of cell wall formation inside the Golgi apparatus of *Thraustochytrium* sp. [49,51], *Schizochytrium* sp. [51], *Diplophrys mutabilis* sp. [35], and *Sorodiplophrys stercorea* sp. [52]. Additionally, it has been shown that the thickness of the outer cell wall negatively correlates with lipid accumulation in *Schizochytrium* sp. ABC101 cells: batch fermentation experiments indicated that nitrogen depletion leads to lipid accumulation, which in turn results in increased cell size and decreased cell wall thickness [53]. In some genera, cell walls can form a contiguous, electron-dense ectoplasmic thread that connects dividing and recently divided cells [48].

The ultrastructure of protists remains largely unexplored. Although members of Labyrinthulea play significant roles in ecological interactions and hold considerable biotechnological value, studies on their cellular organization are currently few and incomplete.

## 4. Metabolic Pathways

For the thraustochytrid species represented in the phylogenetic tree, we examined the available omics data to estimate their enzyme pathways. The references and identifiers of the data with the description of our analysis are provided in Appendix A.3. Eleven such species were identified, nine of which belong to the third clade (Thraustochytrida):

- Three species from the genus *Thraustochytrium*: *T. aureum* ssp. *strugatskii*, *T.* sp. (ATCC 26185), and *T.* sp. LLF1b;
- Three species from the genus *Aurantiochytrium*: *A.* sp. T66, *A.* sp. KH105, and *A. limacinum* sp.;
- Two species from the genus *Schizochytrium*: *S.* sp. (CCTCC M209059), *S. aggregatum* sp. (ATCC 28209);
- One species from the genus *Hondaea*: *H. fermentalgiana* sp.;
- Two species from the fourth clade of the genus *Aplanochytrium*: *A. stocchinoi* sp. and *A. kerguelense* sp.

As a result, the main metabolic pathways and enzymatic components were identified for eleven protist species based on the available data. Metabolic maps of these pathways, along with an assessment of their representation, are provided in Supplementary Figures S5–S21.

**Figure 3:**
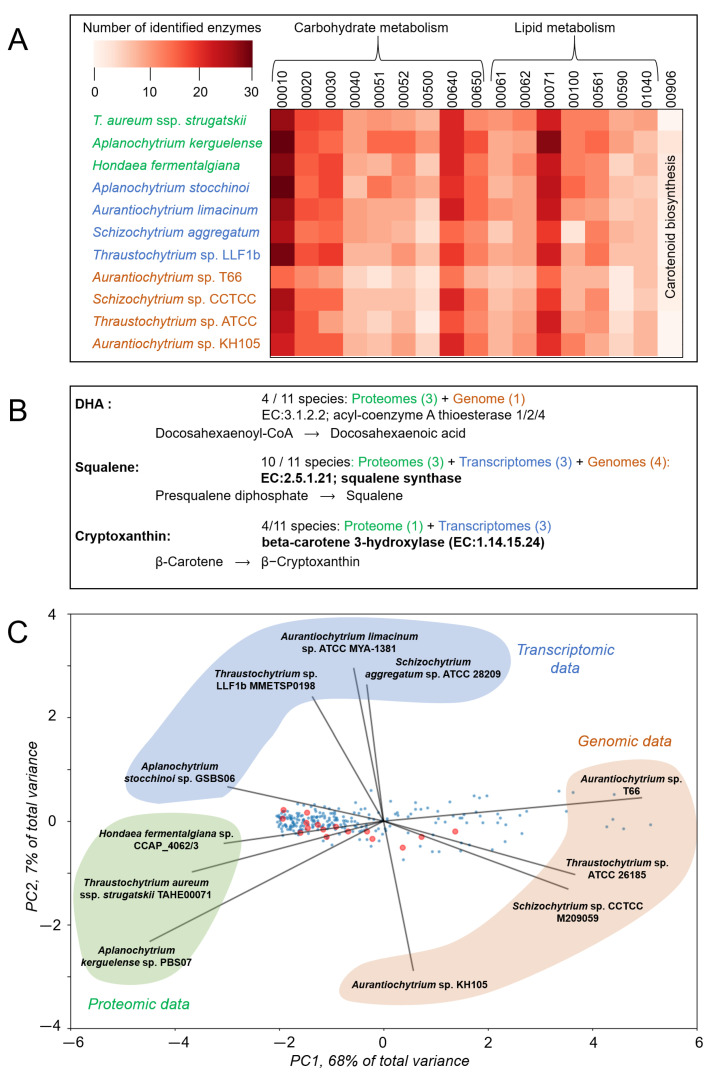
Biochemical characteristics of the eleven thraustochytrids species with available omics data. The colors in sections (**A**–**C**) indicate the data source: green is for data identified through proteomics; blue is for data predicted from transcriptomics; brown is for data predicted from genomics. (**A**) Heatmap showing the representation of enzymatic components in the primary metabolic pathways of interest: carbohydrate metabolism, lipid metabolism, and carotenoid biosynthesis. (**B**) Presence of enzymes involved in the synthesis of docosahexaenoic acid, squalene, and β-cryptoxanthin. (**C**) Clustering of enzymatic pathways based on their representation across different thraustochytrids species in principal components 1 and 2. Red dots represent the primary metabolic pathways of interest from Section (**A**); blue dots represent other metabolic pathways. Black lines show projections of eigenvalues for different thraustochytrids species. Species are grouped by the data type used for biochemical predictions (proteomic, transcriptomic, and genomic). Outliers were removed from the plot.

The high potential of thraustochytrids to produce unsaturated fatty acids, carotenoids, and sterols, particularly DHA and squalene, is well documented [54]. In our analysis of KEGG metabolic pathways, we identified a wide representation of enzymes involved in fatty acid and steroid metabolism and synthesis (Figure 3A): fatty acid biosynthesis (map00061, https://www.kegg.jp/pathway/map00061, accessed on 15 April 2024); fatty acid elongation (map00062, https://www.kegg.jp/pathway/map00062, accessed on 15 April 2024); fatty acid degradation (map00071, https://www.kegg.jp/entry/map00071, accessed on 15 April 2024); steroid biosynthesis (map00100, https://www.kegg.jp/entry/map00100, accessed on 15 April 2024); biosynthesis of unsaturated fatty acids (map01040, https://www.kegg.jp/pathway/map01040, accessed on 15 April 2024).

Among the identified enzymatic components, we found those that are present in more than half of the species (≥6):

1. Fatty acid degradation enzymes (13): frmA, ADH5, adhC (K00121), ALDH (K00128), ACADM, acd (K00249), GCDH, gcdH(K00252), ACAT, atoB (K00626), EHHADH (K07514), CPT1A (K08765), CPT2 (K08766), ACADSB (K09478), ECI1, DCI(K13238), ECI2, PECI (K13239), yiaY (K13954), and ALDH7A1 (K14085);
2. Steroid biosynthesis enzymes (11): DHCR7 (K00213), TM7SF2, ERG24 (K00222), SC5DL, ERG3 (K00227), SMT1, ERG6(K00559), FDFT1 (K00801), LIPA (K01052), CAS1 (K01853), CYP51 (K05917), NSDHL, ERG26 (K07748), CPI1 (K08246), DHCR24, and DWF1 (K09828);
3. Fatty acid biosynthesis enzymes (6): fabD, MCAT, MCT1 (K00645), fabF, OXSM, CEM1 (K09458), ACACA (K11262), CBR4(K11539), HSD17B8 (K13370), and ACSF3 (K18660);
4. Enzymes involved in the elongation and degradation of fatty acids (5): HADH (K00022), ACAA2 (K07508), HADHB(K07509), ECHS1 (K07511), HADHA (K07515);
5. Enzymes involved in fatty acid elongation and biosynthesis of unsaturated fatty acids (4): ELOVL6 (K10203), ELOVL4(K10249), HSD17B12, KAR, IFA38 (K10251), and ACOT7 (K17360);
6. Enzymes involved in fatty acid biosynthesis and degradation (2): ACSL, fadD (K01897), and ACSBG (K15013); enzymes involved in fatty acid degradation and biosynthesis of unsaturated fatty acids (2): ACOX1, ACOX3 (K00232), and ACAA1(K07513); enzyme fatty acid elongation: PPT (K01074); biosynthesis of unsaturated fatty acids (1): SCP2 and SCPX (K08764).

Thraustochytrids can produce ω-3 fatty acids, squalene, and carotenoids; however, DHA remains the only commercial product derived from these organisms [55], and enzymes involved in carotenoid biosynthesis have not yet been described in thraustochytrids.

In four species, we identified enzymes catalyzing the synthesis of eicosapentaenoic acid and docosahexaenoic acid, specifically acyl-coenzyme A thioesterase 1/2/4 [EC:3.1.2.2] (K01068): *Thraustochytrium aureum* ssp. *strugatskii* (TAHE00071), *Thraustochytrium* sp. (ATCC 26185), *Aurantiochytrium* sp. KH105, and *Hondaea fermentalgiana* sp. CCAP_4062/3 (Figure 3B). Many thraustochytrids are known to be capable of synthesizing ω-3 fatty acids, such as *Oblongichytrium* sp. RT2316-13 [56], *Aurantiochytrium*/*Schizochytrium* sp. [57], and *Thraustochytrium aureum* sp. (ATCC 34304) [58]. These findings suggest that not all thraustochytrids species are able to synthesize ω-3 fatty acids. Further proteomic data and a more comprehensive sample of protists are required to accurately assess the presence of this enzyme across the taxon.

In ten species, we identified the squalene biosynthesis enzyme, farnesyl-diphosphate farnesyltransferase [EC:2.5.1.21] (K00801). This may suggest that the capacity to synthesize this important metabolite is widespread among thraustochytrids (Figure 3B).

In the carotenoid synthesis pathway, we identified six enzymes present in thraustochytrids: β-carotene 3-hydroxylase [EC:1.14.15.24] (K15746), which was found in four species: *Aplanochytrium stocchinoi* sp. GSBS06, *Schizochytrium* sp. (CCTCC M209059), *Aurantiochytrium limacinum* sp. (ATCC MYA-1381), and *Hondaea fermentalgiana* sp. CCAP_4062/3 (Figure 3B). We also identified individual components of carotenoid synthesis in single species: carlactone synthase/all-trans-10′-apo-beta-carotenal 13,14-cleaving dioxygenase [EC:1.13.11.69 1.13.11.70] (K17913) in two species (*Aplanochytrium stocchinoi* sp. GSBS06 and *Aplanochytrium kerguelense* sp. PBS07); 9-cis-epoxycarotenoid dioxygenase [EC:1.13.11.51] (K09840) in *Aplanochytrium kerguelense* sp. PBS07; xanthoxin dehydrogenase [EC:1.1.1.288] (K09841) in *Aurantiochytrium* sp. T66; (+)-abscisic acid 8′-hydroxylase [EC:1.14.14.137] (K09843) in *Aplanochytrium kerguelense* sp. PBS07; and torulene dioxygenase [EC:1.13.11.59] (K17842) in *Hondaea fermentalgiana* sp. CCAP_4062/3.

The diversity of metabolic properties in thraustochytrids remains an open question. In the available datasets, the quality of predictions depends strongly on the type of proteomic data used, whether predicted from genomes, transcriptomes, or actual data. This is evident in the clustering of eleven species via principal component analysis (Figure 3C). The principal component values of metabolic processes (with an average number of components ≥5) and eigenvalues of the studied species are provided in Supplementary Table S3. A quantitative representation of enzymatic components is presented in Supplementary Table S4. Notably, the most accurate data on metabolic pathways are available for three species with complete proteomes, which cluster closely in the lower left of the graph. Two species with complete proteomes, *H. fermentagliana* sp. and *T. aureum* ssp. *strugatskii*, belong to the third clade of thraustochytrids in the phylogenetic tree (Figure 1), while the third species, *A. kerguelense* sp., is an outgroup to them and shows a more distinct biochemical profile. This suggests that evolutionary divergence corresponds to the differences in the metabolic characteristics among thraustochytrids.

When using predicted proteomes, the resolution of metabolic maps decreases. For instance, with transcriptome assembly translated into a proteome, approximately 10–20% of enzymatic pathway components are lost compared to real proteomic data, and, with genomic data predictions, 20–50% of such information is lost. Thus, high-quality proteomic data are essential for accurately assessing the presence of specific enzymatic components in thraustochytrids species, although the quantitative distribution of enzymatic components across specific processes remains fairly homogeneous among species (Figure 3C).

It is also worth noting that some enzymatic pathways in protists, such as carotenoid biosynthesis (Figure 3A), are poorly predicted by KEGG pathway data. This may suggest the presence of alternative biochemical reactions in protists for several important metabolic pathways, which could be of economic interest [54,55].

The main limiting factor in clarifying the features of thraustochytrid metabolism is the limited quantity and quality of available omics data. In particular, quantitative and structural proteomics approaches to new enzyme discovery in thraustochytrids, as well as interactomic data to establish interactions between transcription factors and key enzymes in metabolite biosynthesis, may prove especially valuable. An example of such an approach is demonstrated in groundbreaking studies on eukaryotes [59,60]; however, such detailed data are still lacking for protists.

## 5. Culture in Bioreactors

**Figure 4:**
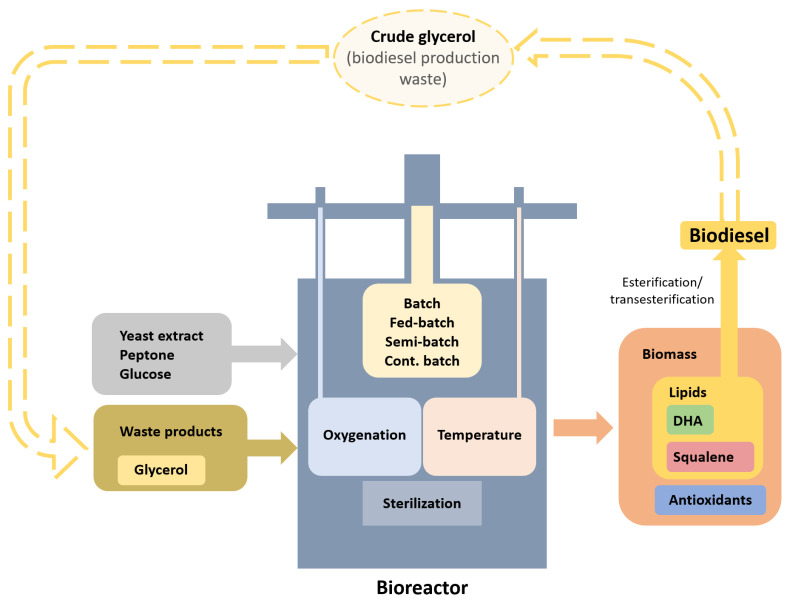
Schematic diagram of a bioreactor for cultivating protists. The diagram illustrates the primary inputs, cultivation conditions, and final products involved in the fermentation.

Thraustochytrids could be used as a food supplement for fish, poultry, and livestock (as reviewed by Wang et al. [61]). They could produce biotechnologically important substances, such as DHA, squalene, β-carotene, and others. Their ability to synthesize a high percentage of lipids may also make them suitable for biodiesel production through lipid esterification and transesterification. Another notable feature is their capacity to utilize various carbon and nitrogen sources for growth, which makes them ideal for waste utilization and serves as an important factor for a sustainable economy. In this section, we discuss various technological advancements that have improved their cultivation in bioreactors. The main points and highlights are summarized in Figure 4 and Supplementary Table S5.

Most studies report the total biomass, lipid content, and DHA accumulation during bioreactor culture (Supplementary Table S5). DHA is an essential product widely used in the food and cosmetics industries. Another important compound synthesized by thraustochytrids is squalene. Currently, squalene is primarily produced from olive oil and shark liver oil [62], but these sources are not economically efficient. Protists could serve as an alternative. Fed-batch cultivation under oxygen-limited conditions has increased squalene production in *Schizochytrium* sp. [63]. Additional studies have reported squalene production in species such as *Schizochytrium* sp. [42] and *Aurantiochytrium* spp. [64,65,66]. Interestingly, adding α-tocopherol enhanced squalene production by 63.2% in *Aurantiochytrium* sp. [67].

Production of carotenoids, including β-carotene, has been reported in *Thraustochytrium* spp. [68,69], *Schizochytrium* spp. [70,71], and *Aurantiochytrium* spp. [66,68,72,73]. Notably, coculturing *Schizochytrium* sp. with *Rhodotorula glutinis* yeasts increased both DHA and β-carotene yields by approximately 1.18 and 1.76-fold, respectively, likely due to a symbiotic relationship [74]. Additional compounds produced by *Aurantiochytrium* spp. include carbohydrates [72,75], astaxanthins [72,76], and arachidonic acid [73].

Biodiesel is produced through the transesterification of vegetable oil or animal fat, resulting in a mixture of different fatty acid methyl esters (FAMEs). Thraustochytrids accumulate high lipid content, making them potential biodiesel precursors. An additional unexpected application for these protists is their ability to utilize glycerol as a carbon source; crude glycerol is a byproduct of conventional biodiesel production. Chen and colleagues demonstrated that crude glycerol can be an efficient carbon source for *Thraustochytrium* sp. Furthermore, they developed a two-step esterification/transesterification process with a lipid conversion rate to FAMEs of up to 91.8% [77]. Other species have also shown promise for biodiesel production, including *Schizochytrium* spp. [78,79] and *Aurantiochytrium* spp. [78,79,80,81,82].

Producing valuable substances requires cost-effective energy sources, and a sustainable economy benefits from repurposing waste products. Thraustochytrids are well suited to meet both needs. Traditionally, yeast extract, peptone, and glucose in artificial seawater are used for cultivation. While these carbon and nitrogen sources may be appropriate for the production of high-value substances such as antioxidants and squalene, they are less ideal for general lipid production or DHA synthesis. Various alternative energy sources have been suggested. For *Thraustochytrium* sp., crude glycerol with acid and CaCl_2_ pretreatment and corn steep liquor have been successfully used [77]. Glycerol has also served as a carbon source in *Schizochytrium* spp. [83,84,85,86,87,88] and *Aurantiochytrium* sp. [89].

In *Schizochytrium* spp., various waste-derived substrates have been explored, such as corn stover hydrolysate for simultaneous β-carotene and DHA production [71], corn steep liquor as a nitrogen source [53], acetic acid [90], waste-derived volatile fatty acids [91], fermentation wastewater [92], fermentation wastewater with microorganism residues [93], saline wastewater from cheese whey demineralization [94], and cane molasses [95]. For *Aurantiochytrium* spp., diverse sources have been utilized, including hydrolyzed cassava pulp [96], anaerobic digestate of agricultural and food industry waste, corn syrup hydrolysate [97], dried Jerusalem artichoke tubers [98], fruit extracts [99], orange peel extract [100], spent osmotic solution from candied fruit production [82], lignocellulosic sugars from Norway spruce [101], organosolv-pretreated Silver birch hydrolysate [64], industrial waste oil [102], inulin following inulinase gene insertion [103], sorghum distillery residue [76], and fish manure from *Takifugu rubripes* [66].

Another approach to enhancing desired product output involves analyzing metabolic pathways and supplementing bottleneck intermediates. For example, adding malate [104] or sesamol and 6-benzylaminopurine [88] increased DHA production in a *Schizochytrium* sp. In an *Aurantiochytrium* sp., monosodium glutamate proved to be an effective nitrogen source for carotenoid and DHA synthesis [73].

Bioreactors (or fermentors) are used to produce large quantities of biomass or final products by utilizing substantial media volumes and enabling control over cultivation parameters. The main types and characteristics of bioreactors have been reviewed elsewhere [105]. Two types of bioreactors (stirred tank and airlift) have been used in the studies on thraustochytrids. It is important to distinguish between cultivation processes: in batch cultivation, cells grow in the initially supplied medium; in fed-batch cultivation, nutrient concentration is kept constant; and in continuous cultivation, the culture medium is continuously replaced. In an airlift bioreactor, medium mixing is achieved through air (gas) bubbles rather than mechanical stirring. Below, we present key technological advancements relevant to thraustochytrids cultivation.

Stirred tank and airlift bioreactors were compared for the cultivation of a *Thraustochytrium* sp., with the airlift bioreactor showing superior results in biomass and DHA production (Chen and Yang, 2018). In a *Schizochytrium* sp., productivity was improved with more efficient oxygenation using a microbubble-type sparger [106]. Aeration is also critical for reducing viscosity caused by extracellular polymeric byproducts [85]. Stepwise aeration has been shown to enhance DHA and carotenoid accumulation in an *Aurantiochytrium* sp. [73]. Interestingly, a constant low oxygen supply can yield better biomass and DHA accumulation than high oxygen levels [89].

Several additional technical innovations have been introduced. Regulating pH during cultivation can sequentially increase biomass and DHA production in a *Schizochytrium* sp. [107]. Continuous three-stage fermentation led to higher DHA production than two-stage fermentation [108], and continuous feeding also proved beneficial [109]. Another technical issue is bioreactor sterilization. While nonaxenic cultivation is feasible for an *Aurantiochytrium* sp. [97], its reproducibility remains uncertain. Chemical sterilization using peracetic acid presents a promising solution for this species [80].

Typically, thraustochytrids are grown without any growth matrix; however, sorghum distillery residue biochar has been suggested as a low-cost growth carrier to enhance *Aurantiochytrium* sp. productivity [76].

Regarding production volume, 12 studies reported data on cultures grown in bioreactors with a volume of 30 L or more (Supplementary Table S5). Notably, an *Aurantiochytrium* sp. was cultured in an 800 L bioreactor [97], and a *Schizochytrium* sp. was cultured in bioreactors with volumes of 5000 L and 35 m^3^ (35,000 L) [110]. These findings indicate that thraustochytrids are well suited for the large-scale production of valuable biotechnological compounds.

## 6. Modeling

The systems biology approach integrates laboratory experiments with mathematical modeling to provide a mechanistic understanding of metabolic pathway functioning and to identify theoretical limitations in various biotechnologically relevant but insufficiently explored organisms. In this section, we examine a range of mathematical methods, including response surface methodology, kinetic modeling, genome-scale metabolic modeling, and artificial neural networks. Key studies and significant highlights for each method are summarized in Supplementary Table S6.

### 6.1. Response Surface Methodology and Artificial Neural Networks

Optimizing culture conditions is essential for enhancing biomass and metabolite production in thraustochytrids. In a study by Rosa et al. (2010) [111], statistical screening experimental designs were employed to identify the key culture variables influencing biomass production in *Aurantiochytrium limacinum* sp. SR21. By combining artificial neural networks, genetic algorithms, and graphical analysis, the study identified optimal levels of these variables, leading to a significant improvement in biomass yield.

Building on these optimization strategies, Zhang et al. (2024) [71] systematically explored the natural carotenoid production capability of a *Schizochytrium* sp., particularly focusing on β-carotene. By enhancing the precursor supply of geranylgeranyl diphosphate, regulating the carbon source through sugar-limited fermentation, and using response surface methodology and artificial neural networks to optimize nitrogen sources, they achieved a 43-fold increase in β-carotene titer, reaching 653.2 mg/L in a 5 L bioreactor.

Together, these studies demonstrate the effectiveness of integrating statistical and machine learning approaches to optimize culture conditions, resulting in significant improvements in both biomass and metabolite production in thraustochytrids.

### 6.2. Kinetic Models

The increasing demand for optimized industrial processes in biotechnology has underscored the value of computer simulations, which provide an efficient way to predict production outcomes with minimal resource and time investment. Simulation-based approaches have proven particularly useful in scaling up complex bioprocesses for high-value compounds, such as DHA and astaxanthin.

Early applications of modeling techniques in bioprocess optimization were demonstrated by Zinnai et al. (2016) [112], who developed a mathematical model to describe the kinetics of supercritical fluid extraction of lipids from a *Schizochytrium* sp. This microorganism, known for its high levels of long-chain polyunsaturated fatty acids (LC-PUFAs), especially DHA, served as a model for optimizing extraction processes for valuable bioactive compounds.

Building on this foundation, Guo et al. (2018) [108] identified growth-uncoupled DHA production in a *Schizochytrium* sp. and developed corresponding kinetic models for fed-batch fermentation. These models accurately described cell growth, substrate utilization, and DHA production, leading to the predictive framework development for multi-stage continuous fermentation. The model’s predictions were validated in laboratory-scale bioreactors, enabling optimized parameters for two-stage and three-stage continuous fermentation processes.

Further expanding on DHA production, Contreras et al. (2021) [113] investigated fed-batch fermentation in *Schizochytrium limacinum* sp. OUC88. Using MATLAB, Contreras developed a kinetic model that simulated DHA concentrations of 150 g/L in fed-batch mode, compared to just 30 g/L in batch mode. The study emphasized the efficiency of fed-batch fermentation for DHA production and highlighted the potential for further optimization.

Advancements in fed-batch optimization were subsequently made by Rohman et al. (2022) [114], who employed multiobjective optimal control techniques to balance competing goals in DHA production. The study utilized the ϵ constraint method and the nondominated sorting genetic algorithm (NSGA-II) to generate Pareto front solutions, optimizing feed flow rates to minimize processing time while maximizing DHA yield. In a related study, Rohman et al. (2022) [115] refined feeding strategies using enhanced scatter search and control vector parametrization, identifying optimal trajectories to maximize DHA concentration, reduce processing time, and improve economic viability.

In further contributions to DHA production scalability, Du et al. (2022) [110] developed a kinetics-integrated computational fluid dynamics (CFD) model that facilitated the scale-up of DHA fermentation. This model, which combined biokinetics with CFD to simulate biomass growth, lipid accumulation, and flow dynamics in a 5 L bioreactor, was successfully scaled up to a 35 m^3^ bioreactor. The process achieved high yields, including 99.2 g/L biomass, 55.7 g/L lipid concentration, and 52.5% DHA content, showcasing the potential of CFD–kinetic models for efficient large-scale DHA production.

Recently, Xiao et al. (2024) [116] expanded the application of kinetic modeling to include a broader range of intracellular products. In a study on the marine protist *Thraustochytrium striatum* sp., Xiao developed a kinetic model that accounted for substrate consumption, cell growth, and the accumulation of lipids and astaxanthin. This work broadens the scope of kinetic modeling in microbial production systems, providing valuable insights for future applications.

Together, these studies demonstrate the power of kinetic and CFD modeling in optimizing bioprocesses for valuable bioproducts. From supercritical fluid extraction to fed-batch and continuous fermentation, these models establish a robust framework for enhancing productivity, scalability, and economic efficiency in industrial biotechnology.

### 6.3. Genome-Scale Metabolic Models

Genome-scale metabolic models (GEMs) have become foundational in systems biology for modeling metabolic processes, offering insights into the functioning and theoretical limitations of various biotechnologically relevant organisms [117]. GEMs provide a mathematical representation of an organism’s complete biochemical pathways based on genomic information, allowing researchers to simulate metabolic behavior and optimize production outcomes [118].

Constructing a GEM involves several key steps: (a) draft model generation, (b) model refinement and curation, (c) metabolic network formation, and (d) network analysis. During the initial phase, genome annotation, the functional annotation of genes and proteins, and the assembly of metabolic reactions are essential. The second phase involves verifying gene–protein–reaction associations, estimating biomass components, incorporating transport and exchange reactions, and addressing network gaps. In the third phase, the curated network is converted into a computable format (e.g., SBML, XML, and MAT), model constraints are defined, and the objective function is set. Finally, flux balance analysis is used to validate the model’s accuracy by examining growth rates, substrate utilization, and product formation through metabolic flux analysis across pathways [119].

The first GEM for thraustochytrids, iCY1170_DHA, was constructed for the well-studied strain *Schizochytrium limacinum* sp. SR21 [120]. Ye and colleagues [120] developed this model by integrating data from three existing models, enabling accurate subcellular compartmentalization predictions and implementing specific model refinements. Their study also proposed strategies for optimizing cultivation conditions and strain engineering to enhance DHA yield.

Subsequently, iCS1079 was developed for *Oblongichytrium* sp. RT2316-13 by modifying iCY1170_DHA to reflect RT2316-13’s metabolic pathways [121]. This GEM was paired with an additional mathematical model to simulate fermentation profiles in batch cultures, showing good agreement with experimental data. Notably, the model effectively predicted the impact of oxygen availability on lipid synthesis rates.

Following this, a high-quality GEM, iVS1191 [122], was reconstructed for *Aurantiochytrium* sp. T66. Through iterative rounds of refinement and extensive manual curation, this model expanded the metabolic scope and achieved stoichiometric consistency, metabolic connectivity, and robust annotations. iVS1191 demonstrated high concordance with experimental growth data, accurately predicting in vivo growth rates on minimal carbon media. This model provides a solid foundation for metabolic engineering and process optimization strategies, particularly for enhancing ω-3 PUFA production in silico.

The most recent and advanced model, iCS648 [123], was developed for *Thraustochytrium* sp. RT2316-16. This model examined the effects of individual amino acids as the sole nitrogen source on biomass and lipid production. Based on an annotated genome, iCS648 employed flux balance analysis to interpret nutrient consumption and growth patterns, with experimentally derived parameters used to constrain nutrient flux and biomass-specific growth rates. The model identified a relationship between ATP production for maintenance and glucose consumption, which was then used in linear optimization to predict specific growth rates under various conditions.

Collectively, these GEMs represent progressive advancements in understanding and optimizing the metabolic pathways of thraustochytrids. They provide a valuable framework for exploring metabolic engineering strategies and optimizing production processes for high-value compounds such as DHA and ω-3 PUFAs.

## 7. Future Perspectives

The expanding field of thraustochytrids research presents numerous opportunities for advancing biotechnological applications. This review highlighted the remarkable capacity of thraustochytrids to convert diverse metabolites into valuable lipid biochemicals through recycling processes in bioreactors. Future research should focus on optimizing these processes, particularly by refining conditions affecting fatty acid production through mathematical modeling and the use of available omics data. Rather than emphasizing a single direction, we underscore several areas discussed in this review that warrant further attention.

In particular, the current 18S rRNA data for various types of these protists provide a basis for a consistent classification within the framework of basal speciation events. However, closely related protist species are challenging to distinguish using 18S rRNA alone, and a more detailed understanding of interspecific relationships may require full-genome data, which are currently insufficient to reconstruct a comprehensive evolutionary picture for this taxon.

Further research is needed to investigate the ultrastructure, functional properties, and growth regulation of organelles such as lipid bodies, ectoplasmic nets, and scales throughout the protist life cycle.

While available omics data offer a general overview of the enzymatic components involved in the biosynthesis of various secondary metabolites in protists, some enzymes responsible for certain metabolites, such as carotenoids, remain unidentified. Additional in-depth studies of the protist metabolome and enzyme systems are essential to fill these knowledge gaps.

Various metabolites can be used as nutrients for culturing protists in bioreactors; however, the digestibility of different substrates varies among protists. Additional experiments and mathematical modeling approaches are needed to optimize these processes. Here, new detailed omics data could be invaluable, clarifying the presence or absence of specific enzymatic components necessary for the biosynthesis of target compounds or the efficient digestion of substrates.

Most current mathematical models of protist metabolism rely on response surface methodology. However, genome-scale metabolic modeling offers a more precise and powerful approach and should be further explored to enhance our understanding and optimization of protist metabolism.

## Data Availability

Data used in this study are available in the Supplementary Files. Other additional materials and results of our calculations can be provided to those interested upon reasonable request.

## Supplementary Materials

The following supporting information can be downloaded at: https://www.mdpi.com/article/10.3390/ijms252313172/s1. References [124,125,126,127,128,129,130,131,132,133,134,135,136,137,138,139,140,141,142,143,144,145] are cited in the Supplementary Table S2.

## Abbreviations

The following abbreviations are used in this manuscript:

rRNA	ribosomal RNA
ω-3 PUFAs	long-chain, omega-3 polyunsaturated fatty acids
FAMEs	fatty acid methyl esters
EPA	eicosapentaenoic acid
DHA	docosahexaenoic acid

## Appendix A. Procedure for Selecting Materials and Articles for Review

### Appendix A.1. Phylogenetics Articles

The nucleotide sequences for the Bigyra taxon were retrieved using the query taxid:2683628[organism:exp] 18S

The sequence AB022110.1 (18S rRNA *Thraustochytrium aureum*) was identified. A search for homologs based on the AB022110.1 sequence (18S rRNA *Thraustochytrium aureum*) was conducted of the 18S database, excluding the *Blastocystis* taxon, using the BLAST algorithm in the NCBI database (https://blast.ncbi.nlm.nih.gov/Blast.cgi, accessed on 15 April 2024) [146]. A total of 3680 homologous sequences were found. Sequences with generic names not classified under Labyrinthulea were removed, resulting in a final set of 2914 sequences.

Next, multiple alignment of the sequence data was performed using MAFFT software (version 7, https://mafft.cbrc.jp/alignment/server/, accessed on 15 April 2024) [147] with the following parameters: FFT-NS-2 algorithm, scoring matrix for nucleotide sequences set to 200 PAM/K = 2, gap opening penalty of 1.53, and nucleotide sequence orientation adjusted to the first sequence. Sequences containing more than 90% gaps were removed from the alignment. An expert assessment of domains was then conducted, and sequences with gaps in conserved domains of 18S rRNA were excluded. After this two-step filtering, the final dataset included 569 sequences from Labyrinthulea class representatives and several outgroup sequences.

This alignment was used to construct a phylogenetic tree using the SAMEM v. 0.83 web server (http://pixie.bionet.nsc.ru/samem/, accessed on 15 April 2024) [148]. The maximum likelihood algorithm was chosen with 20 rate categories. The Archaeopteryx program (https://www.phylosoft.org/archaeopteryx/, accessed on 15 April 2024) was used to visualize the phylogenetic tree; branches with bootstrap support below 0.6 were collapsed.

### Appendix A.2. Ultrastructure Articles

Based on the phylogenetic analysis we selected 25 genera for article search: *Adriamonas*, *Amphifilidae*., *Amphitrema*, *Aplanochytrium*, *Archerella*, *Aurantiochytrium*, *Botryochytrium*, *Diplophrys*, *Hondaea*, *Japonochytrium*, *Labyrinthula*, *Labyrinthulochytrium*, *Labyrinthuloides*, *Monorhizochytrium*, *Mucochytrium*, *Oblongichytrium*, *Parietichytrium*, *Phycophthorum*, *Schizochytrium*, *Sicyoidochytrium*, *Sorodiplophrys*, *Stellarchytrium*, *Thraustochytriidae*, *Thraustochytrium*, and *Ulkenia*. We used the Google Scholar search engine (https://scholar.google.com/, accessed on 15 April 2024) with the following parameters: “[species name] + electron microscopy + ultrastructure”,

Search results for articles numbered 1–50 were reviewed without applying a time filter. Based on the search, no articles with ultrastructural data were found for the following species: *Amphitrema* sp., *Archerella* sp., *Botryochytrium* sp., *Hondaea* sp., *Japonochytrium* sp., *Labyrinthuloides* sp., *Monorhizochytrium* sp., *Mucochytrium* sp., *Parietichytrium* sp., *Sicyoidochytrium* sp., *Stellarchytrium* sp., and *Thraustochytriidae* sp. All retrieved articles were analyzed for ultrastructural data on parameters including cell stage, bothrosome/sagenetosome, lipid bodies, ectoplasmic net, scales, paranuclear bodies, and electron-dense bodies.

In each case, a quick search was conducted using these parameters. If a parameter was not identified, additional searches were performed using synonyms and partial terms. For example, “scales” were also searched as “cell wall”, and “paranuclear bodies” were verified by searching for terms containing “nucl”. This search and data processing resulted in 42 articles with ultrastructural features, summarized in Supplementary Table S2.

### Appendix A.3. Metabolic Pathways

To evaluate the metabolic features, we searched public repositories for available omics data on thraustochytrids and identified eleven species with such data.

In particular, genomes, transcriptomes, and proteomes were available for three species:

*Aplanochytrium kerguelense* sp. PBS07; *Hondaea fermentalgiana* sp. CCAP_4062/3 [149]; *Thraustochytrium aureum* ssp. *strugatskii* (TAHE00071) [20]. Genomic data were available for four additional species:

*Aurantiochytrium* sp. T66 (GCA_001462505.1) [150]; *Schizochytrium* sp. (CCTCC M209059) (GCA_000818945.1) [151]; *Thraustochytrium* sp. (ATCC 26185) (GCA_002154235.1) [152]; *Aurantiochytrium* sp. KH105 (GCA_003116975.1) [153]. Transcriptomic data were available for four additional species:

*Aplanochytrium stocchinoi* sp. GSBS06 (SRX549055) [154]; *Aurantiochytrium limacinum* sp. (ATCC MYA-1381) (SRX554098) [154]; *Schizochytrium aggregatum* sp. (ATCC 28209) (SRX554217) [154]; *Thraustochytrium* sp. LLF1b MMETSP0198 (SRX551253) [154]. To compare metabolic pathways, transcriptomic and genomic data were used to predict the proteomes for eight of these protist species. For the four species with available raw transcriptome data from the Marine Microbes and Eukaryotes Transcriptome Sequencing Project (SRX549055, SRX554098, SRX554217, and SRX551253), transcripts were assembled using Trinity v. 2.15 [155], and the assembled transcripts were used to predict corresponding proteins with TransDecoder v5.7.0. For the four species with available genomes (GCA_001462505.1, GCA_000818945.1, GCA_002154235.1, and GCA_003116975.1), proteomes were predicted using AUGUSTUS v. 3.5.0 [156].

The predicted proteomes for these eleven thraustochytrids species were then analyzed to identify the present enzymes using the BlastKOALA web service v. 3.0 [157]. The resulting annotations were visualized with the KEGG mapper [158].

### Appendix A.4. Bioreactor and Modeling Articles

Based on phylogenetic analysis, we selected six species for article search: an *Adriamonas* sp., an *Aurantiochytrium* sp., a *Japonochytrium* sp., a *Schizochytrium* sp., a *Thraustochytrium* sp., and a *Ulkenia* sp. We used the Google Scholar search engine (https://scholar.google.com/, accessed on 15 April 2024) with the following parameters: “[species name] + bioreactor”, filtered by the years 2018–2024, with results limited to the top 1–50 entries. There were no articles on *Ulkenia* spp. culture in bioreactors. We focused on bioreactor usage and excluded articles earlier than 2018. Among the 79 articles, we excluded some as there was no bioreactor cultivation, and others lacked substantial novelty. This gave us 52 articles, which summarized in Supplementary Table S5.

For the modeling section, we used the first 20 links from the Google Scholar search engine (https://scholar.google.com/, accessed on September 2024) with the following parameters: “[species name] + model”.
