# Supplementary material for: Thraustochytrids: Evolution, Ultrastructure, Biotechnology, and Modeling
Source: Int J Mol Sci. 2024 Dec 7;25(23):13172. doi: 10.3390/ijms252313172 (PMC11642839; doi:10.3390/ijms252313172)
Supplement: Supplementary file 1 [file ijms-25-13172-s001.zip › Supplementary figure captions.pdf]

Supplementary figure S1. Phylogenetic tree of the Clade I. E – ectoplasmic network, B – bothrosome.

Supplementary figure S2. Phylogenetic tree of the Clade II. E – ectoplasmic network, B – bothrosome.

Supplementary figure S3. Phylogenetic tree of the Clade III. E – ectoplasmic network, B – bothrosome.

Supplementary figure S4. Phylogenetic tree of the Clade IV. E – ectoplasmic network, B – bothrosome.

Supplementary figure S5. Heatmap of identified enzymatic components of KEGG PATHWAY: map00010 (“GLYCOLYSIS / GLUCONEOGENESIS”) among thraustochytrids.

Supplementary figure S6. Heatmap of identified enzymatic components of KEGG PATHWAY: map00020 (“CITRATE CYCLE (TCA CYCLE)”) among thraustochytrids.

Supplementary figure S7. Heatmap of identified enzymatic components of KEGG PATHWAY: map00030 (“PENTOSE PHOSPHATE PATHWAY”) among thraustochytrids.

Supplementary figure S8. Heatmap of identified enzymatic components of KEGG PATHWAY: map00040 (“PENTOSE AND GLUCURONATE INTERCONVERSIONS”) among thraustochytrids.

Supplementary figure S9. Heatmap of identified enzymatic components of KEGG PATHWAY: map00051 (“FRUCTOSE AND MANNOSE METABOLISM”) among thraustochytrids.

Supplementary figure S10. Heatmap of identified enzymatic components of KEGG PATHWAY: map00052 (“GALACTOSE METABOLISM”) among thraustochytrids.

Supplementary figure S11. Heatmap of identified enzymatic components of KEGG PATHWAY: map00061 (“FATTY ACID BIOSYNTHESIS”) among thraustochytrids.

Supplementary figure S12. Heatmap of identified enzymatic components of KEGG PATHWAY: map00062 (“FATTY ACID ELONGATION”) among thraustochytrids.

Supplementary figure S13. Heatmap of identified enzymatic components of KEGG PATHWAY: map00071 (“FATTY ACID DEGRADATION”) among thraustochytrids.

Supplementary figure S14. Heatmap of identified enzymatic components of KEGG PATHWAY: map00100 (“STEROID BIOSYNTHESIS”) among thraustochytrids.

Supplementary figure S15. Heatmap of identified enzymatic components of KEGG PATHWAY: map00500 (“STARCH AND SUCROSE METABOLISM”) among thraustochytrids.

Supplementary figure S16. Heatmap of identified enzymatic components of KEGG PATHWAY: map00561 (“GLYCEROLIPID METABOLISM”) among thraustochytrids.

Supplementary figure S17. Heatmap of identified enzymatic components of KEGG PATHWAY: map00590 (“ARACHIDONIC ACID METABOLISM”) among thraustochytrids.

Supplementary figure S18. Heatmap of identified enzymatic components of KEGG PATHWAY: map00640 (“PROPANOATE METABOLISM”) among thraustochytrids.

Supplementary figure S19. Heatmap of identified enzymatic components of KEGG PATHWAY: map00650 (“BUTANOATE METABOLISM”) among thraustochytrids.

Supplementary figure S20. Heatmap of identified enzymatic components of KEGG PATHWAY: map00906 (“CAROTENOID BIOSYNTHESIS”) among thraustochytrids.

Supplementary figure S21. Heatmap of identified enzymatic components of KEGG PATHWAY: map01040 (“BIOSYNTHESIS OF UNSATURATED FATTY ACIDS”) among thraustochytrids.
