# Supplementary figures and images for: Thraustochytrids: Evolution, Ultrastructure, Biotechnology, and Modeling
Source: Int J Mol Sci. 2024 Dec 7;25(23):13172. doi: 10.3390/ijms252313172 (PMC11642839; doi:10.3390/ijms252313172)

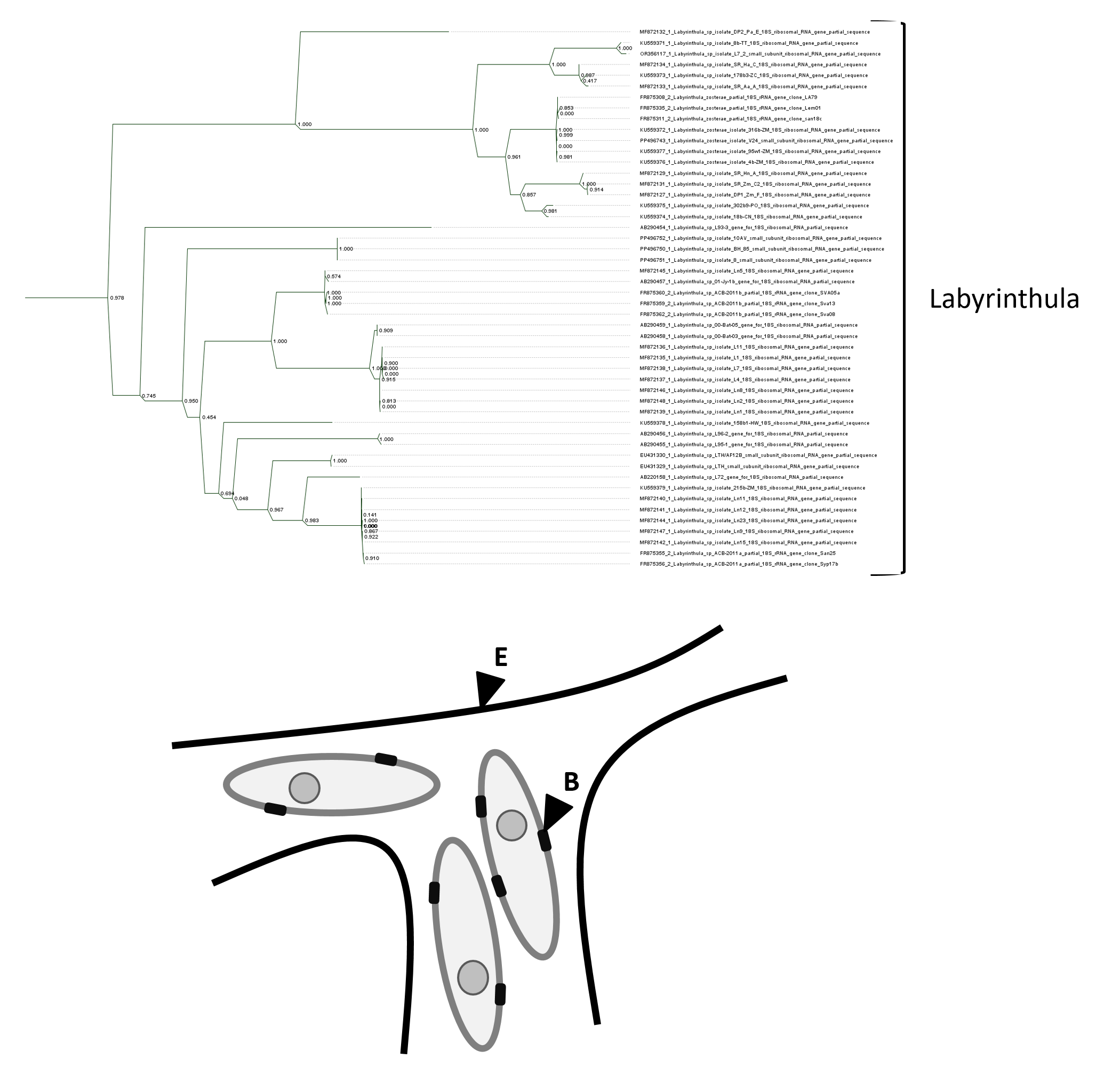

Supplement: Supplementary file 1 [file ijms-25-13172-s001.zip › Supplementary figure S1.tif]

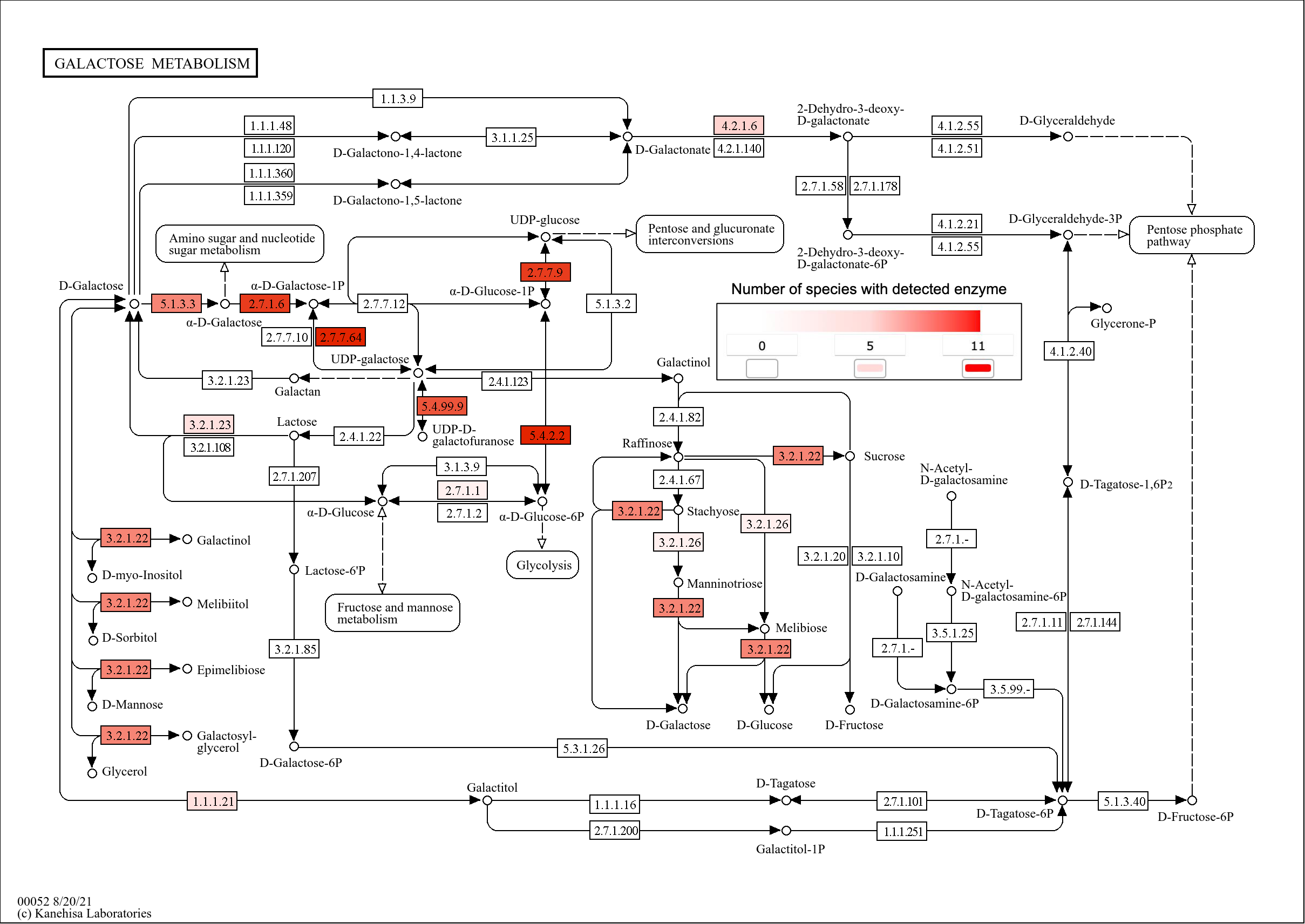

Supplement: Supplementary file 1 [file ijms-25-13172-s001.zip › Supplementary figure S10_map00052@2x_20240919_142427.png]

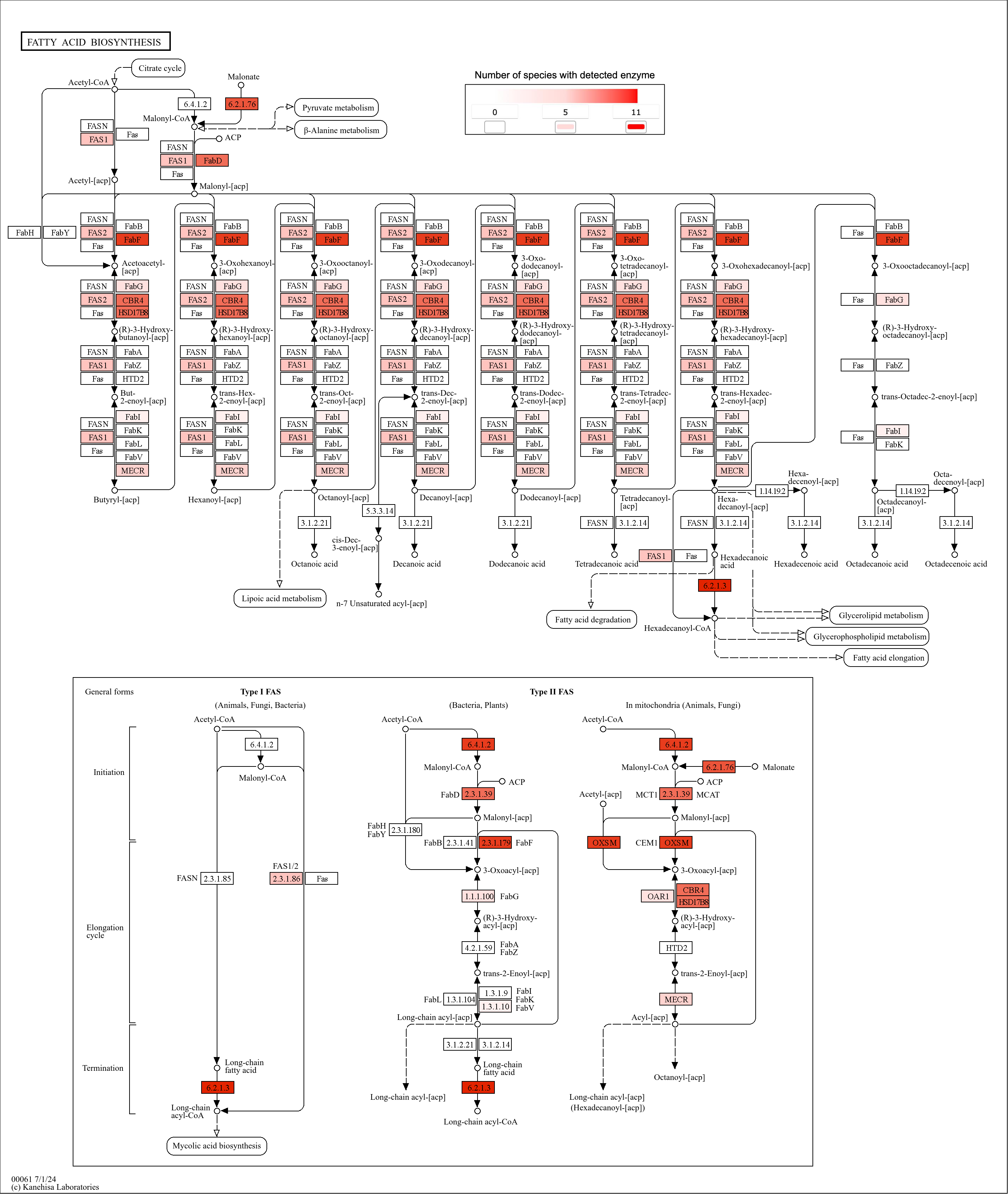

Supplement: Supplementary file 1 [file ijms-25-13172-s001.zip › Supplementary figure S11_map00061@2x_20240919_133924.png]

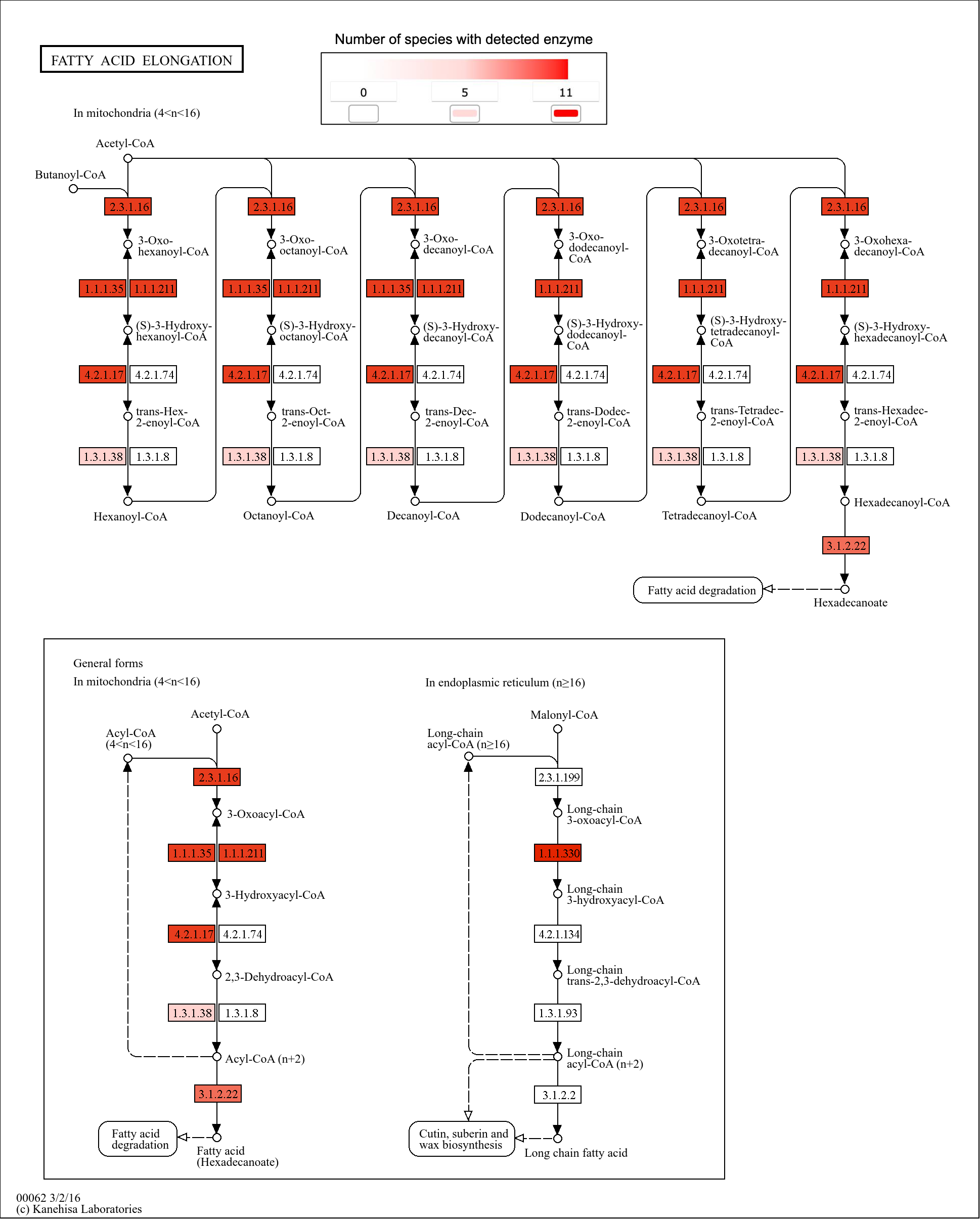

Supplement: Supplementary file 1 [file ijms-25-13172-s001.zip › Supplementary figure S12_map00062@2x_20240923_164840.png]

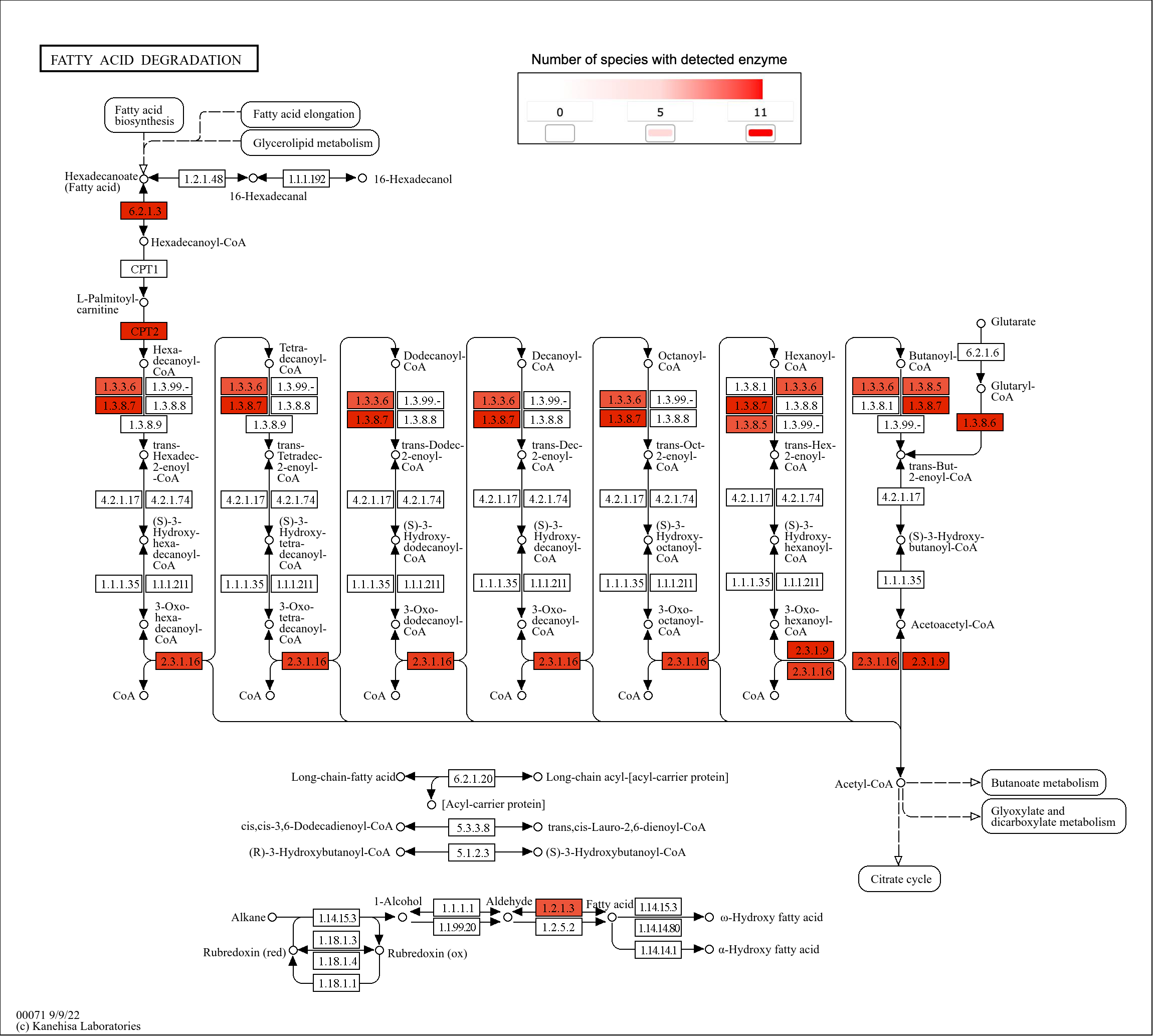

Supplement: Supplementary file 1 [file ijms-25-13172-s001.zip › Supplementary figure S13_map00071@2x_20240923_164941.png]

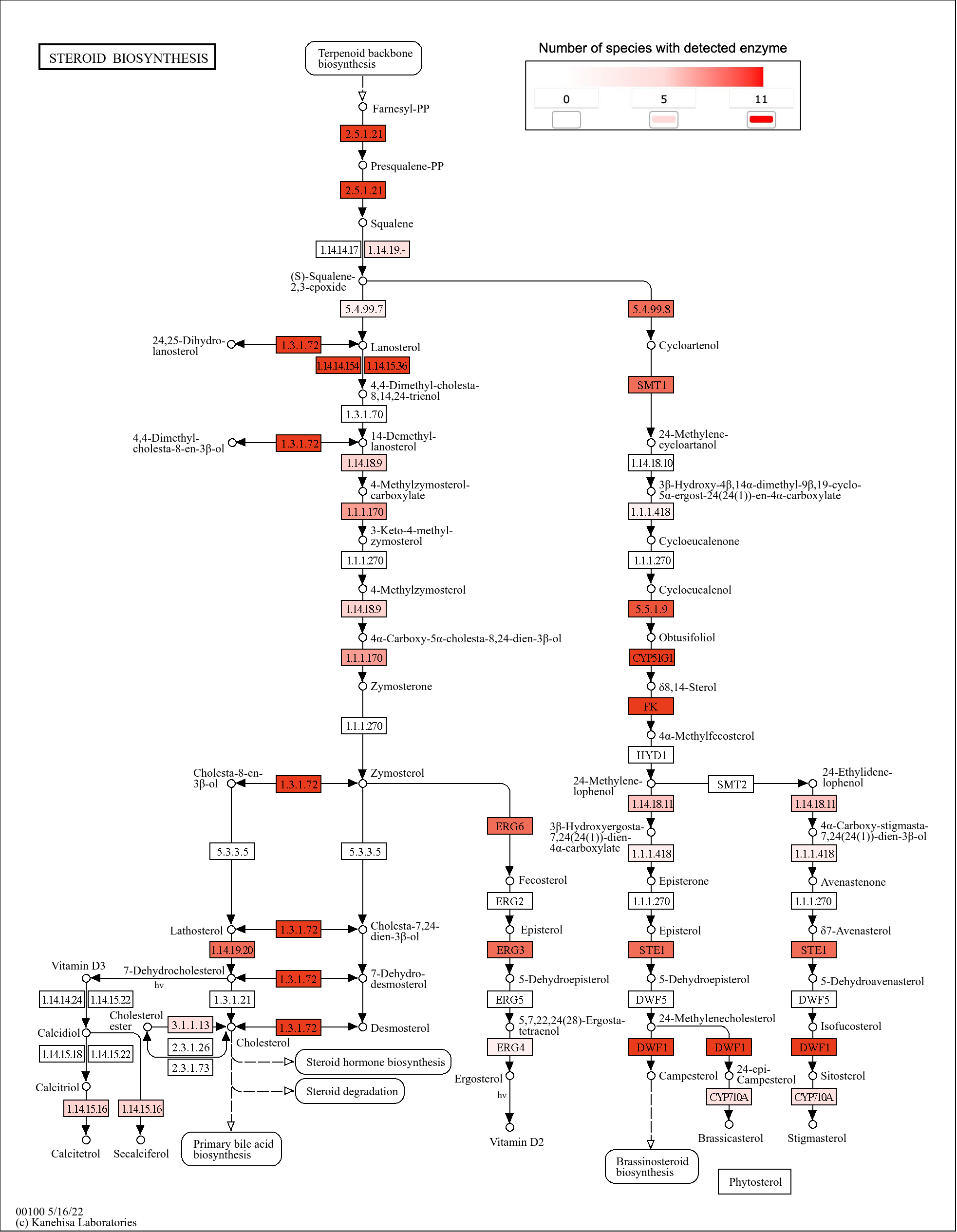

Supplement: Supplementary file 1 [file ijms-25-13172-s001.zip › Supplementary figure S14_map00100@2x_20240919_133430.png]

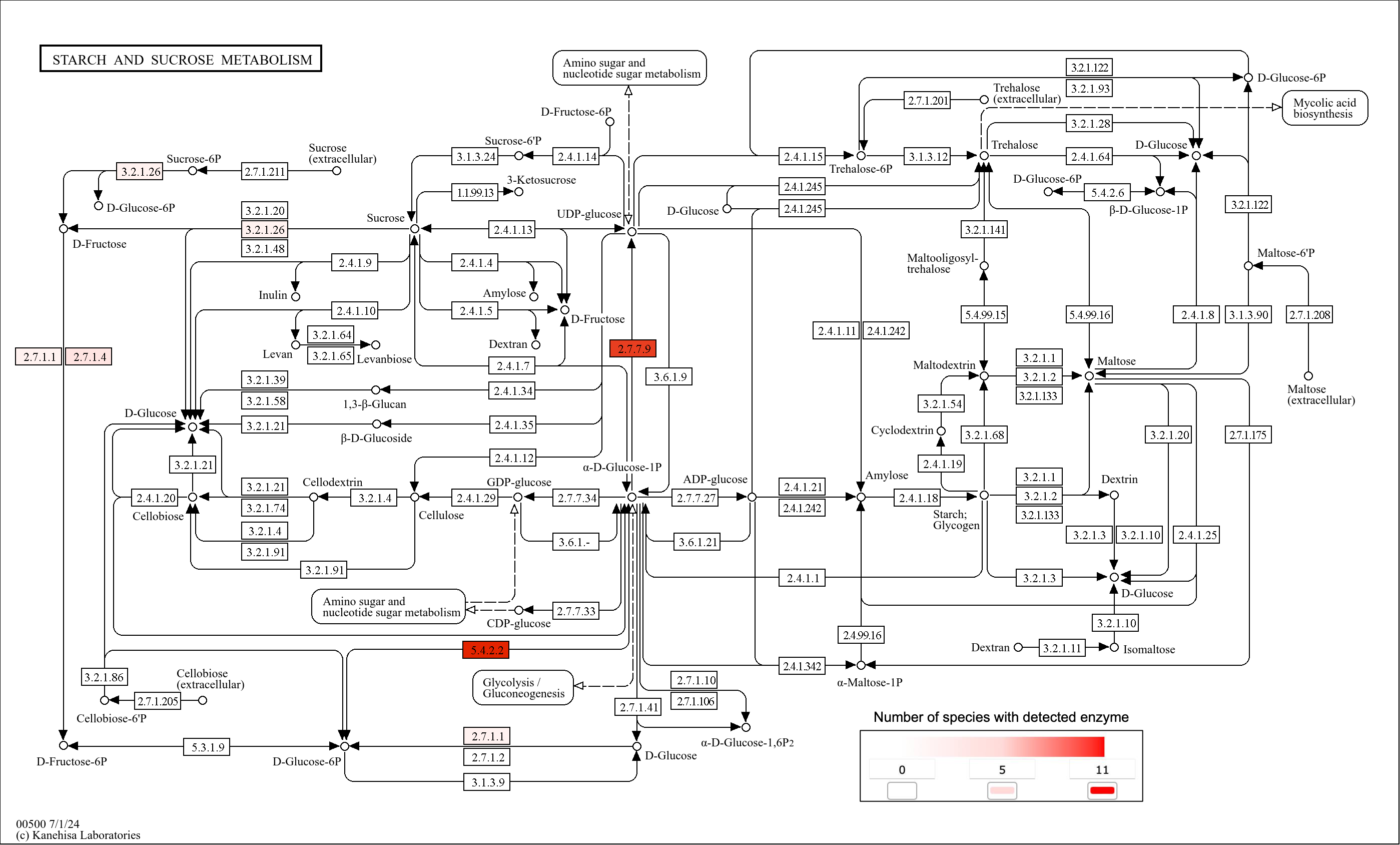

Supplement: Supplementary file 1 [file ijms-25-13172-s001.zip › Supplementary figure S15_map00500@2x_20240923_165038.png]

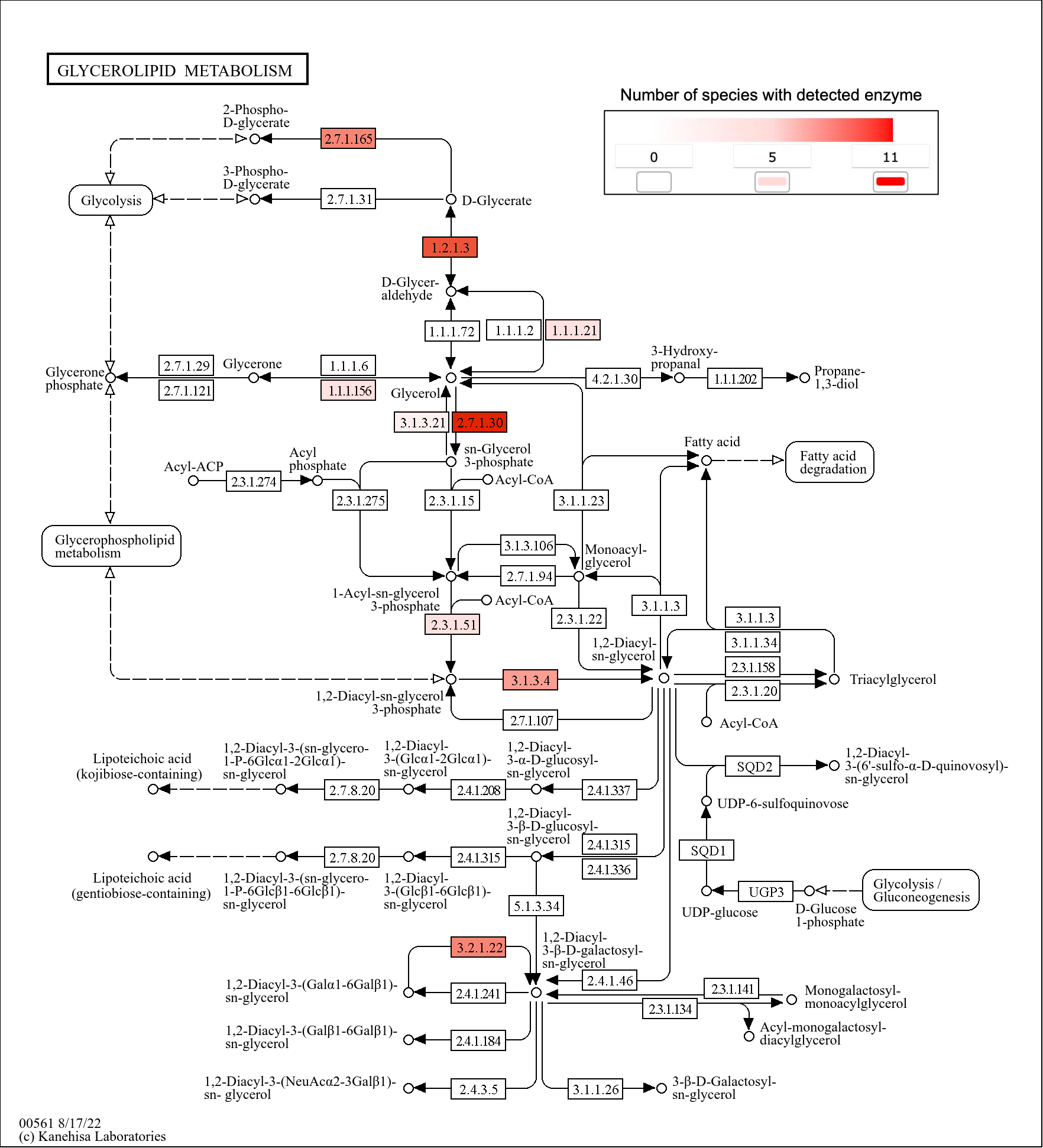

Supplement: Supplementary file 1 [file ijms-25-13172-s001.zip › Supplementary figure S16_map00561@2x_20240923_165130.png]

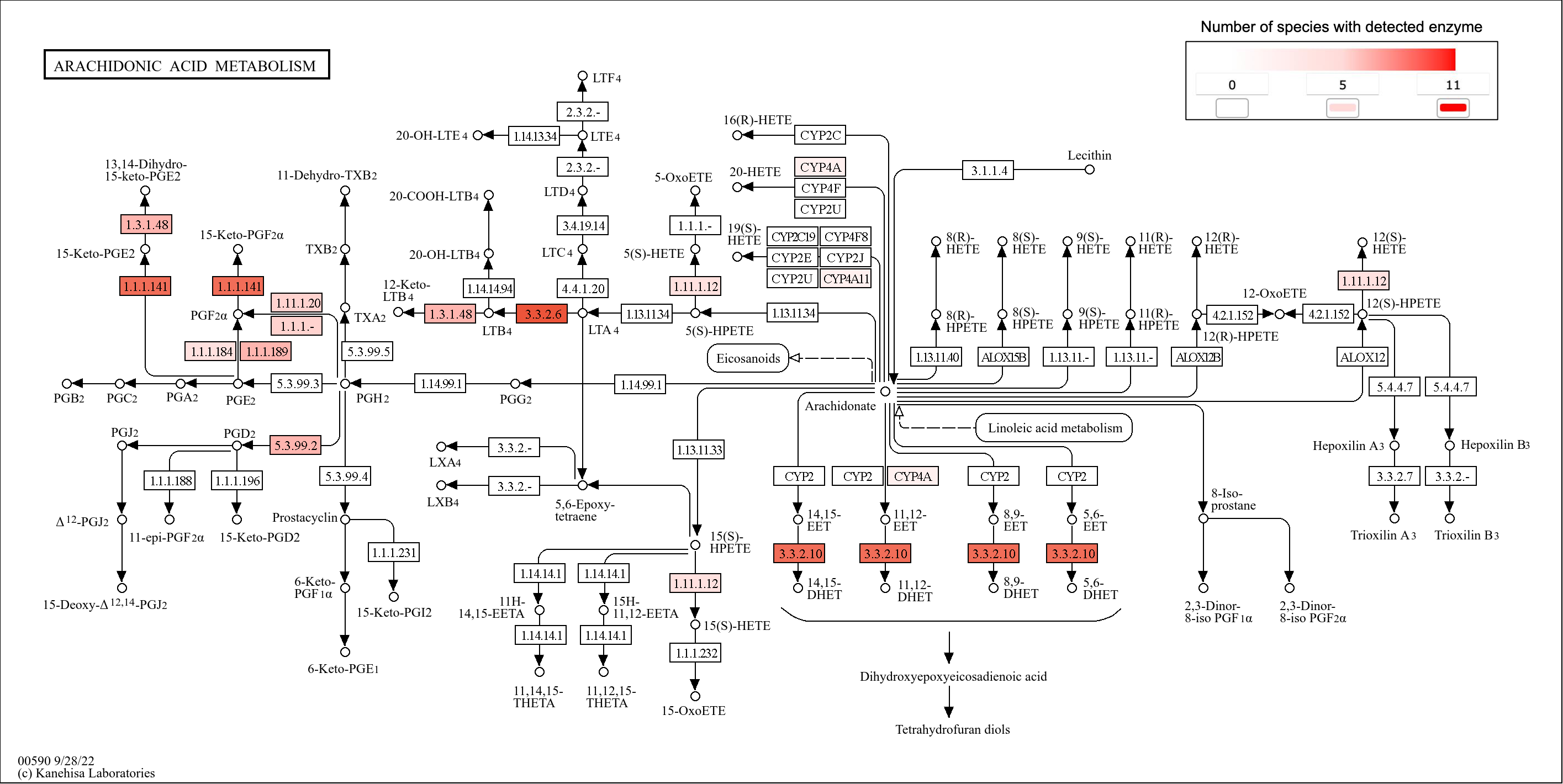

Supplement: Supplementary file 1 [file ijms-25-13172-s001.zip › Supplementary figure S17_map00590@2x_20240923_165213.png]

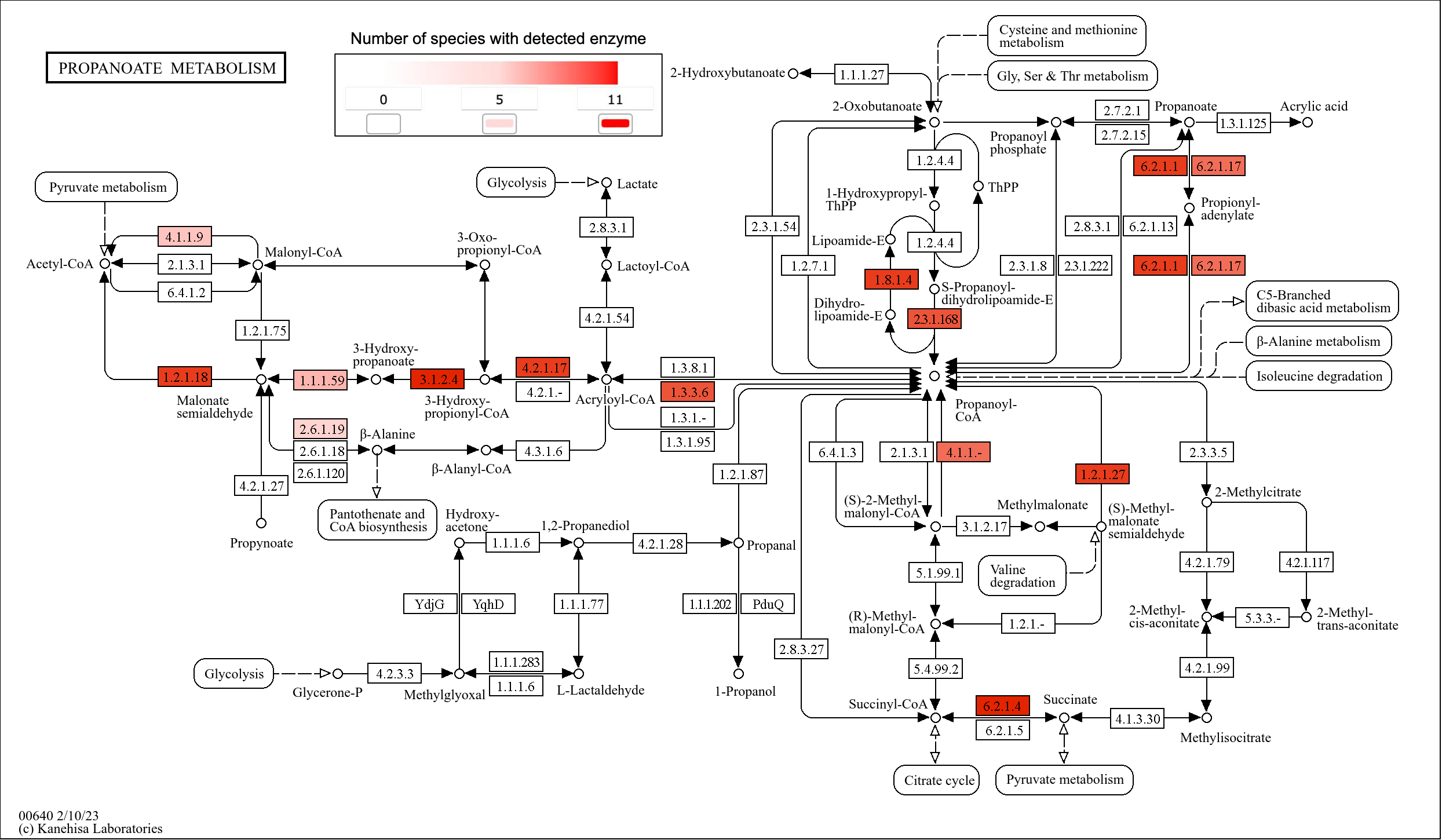

Supplement: Supplementary file 1 [file ijms-25-13172-s001.zip › Supplementary figure S18_map00640@2x_20240923_165257.png]

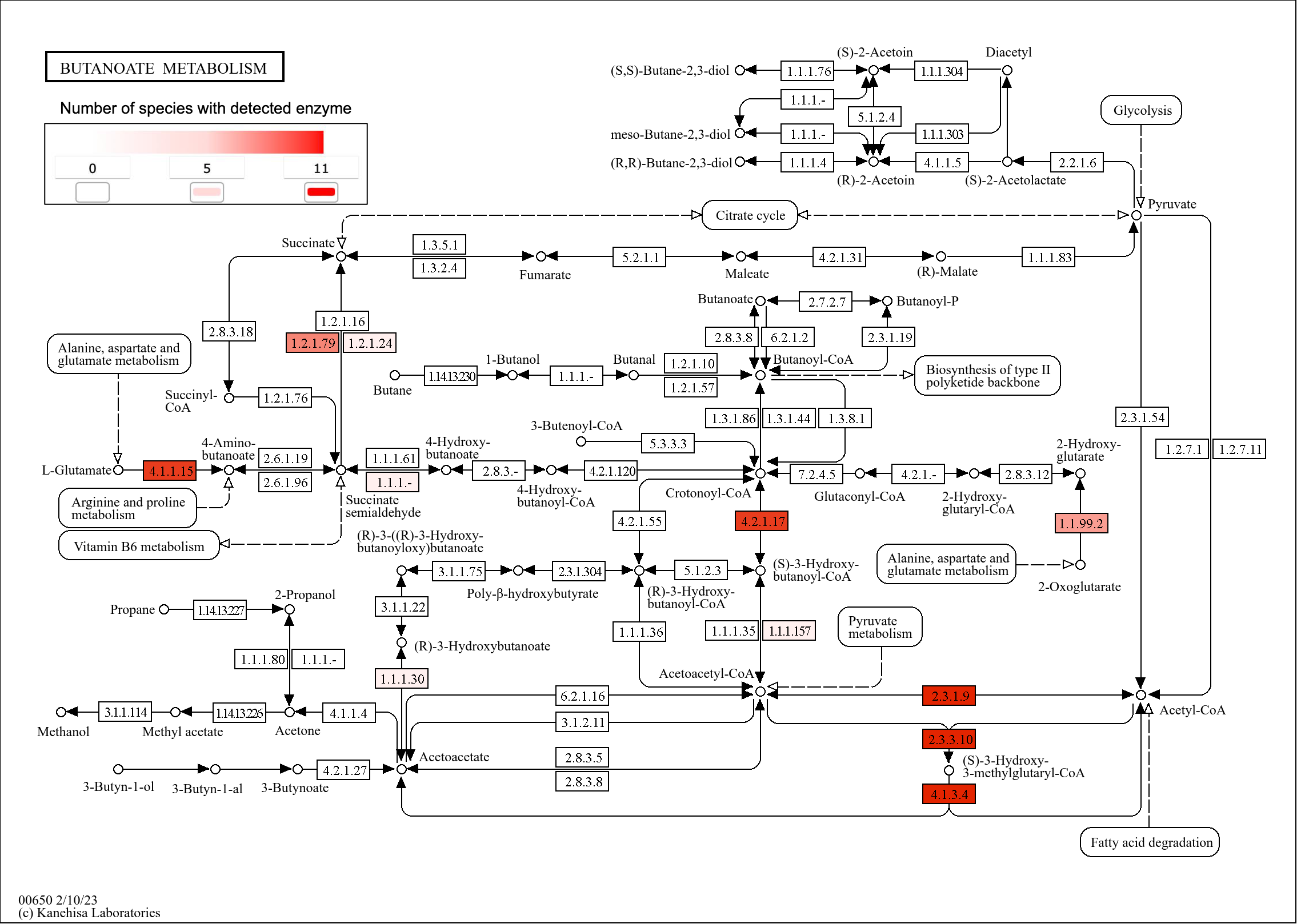

Supplement: Supplementary file 1 [file ijms-25-13172-s001.zip › Supplementary figure S19_map00650@2x_20240923_165346.png]

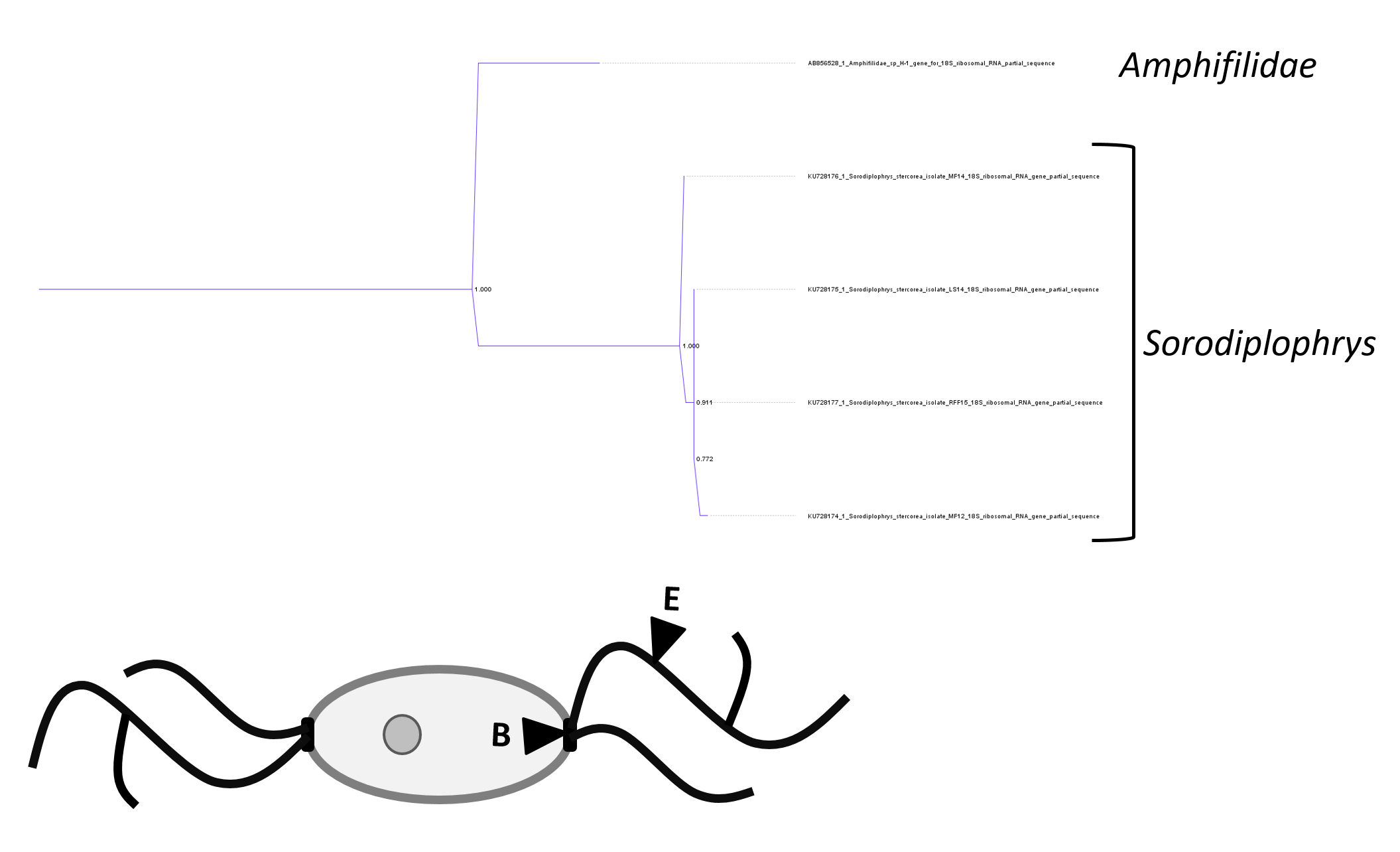

Supplement: Supplementary file 1 [file ijms-25-13172-s001.zip › Supplementary figure S2.tif]

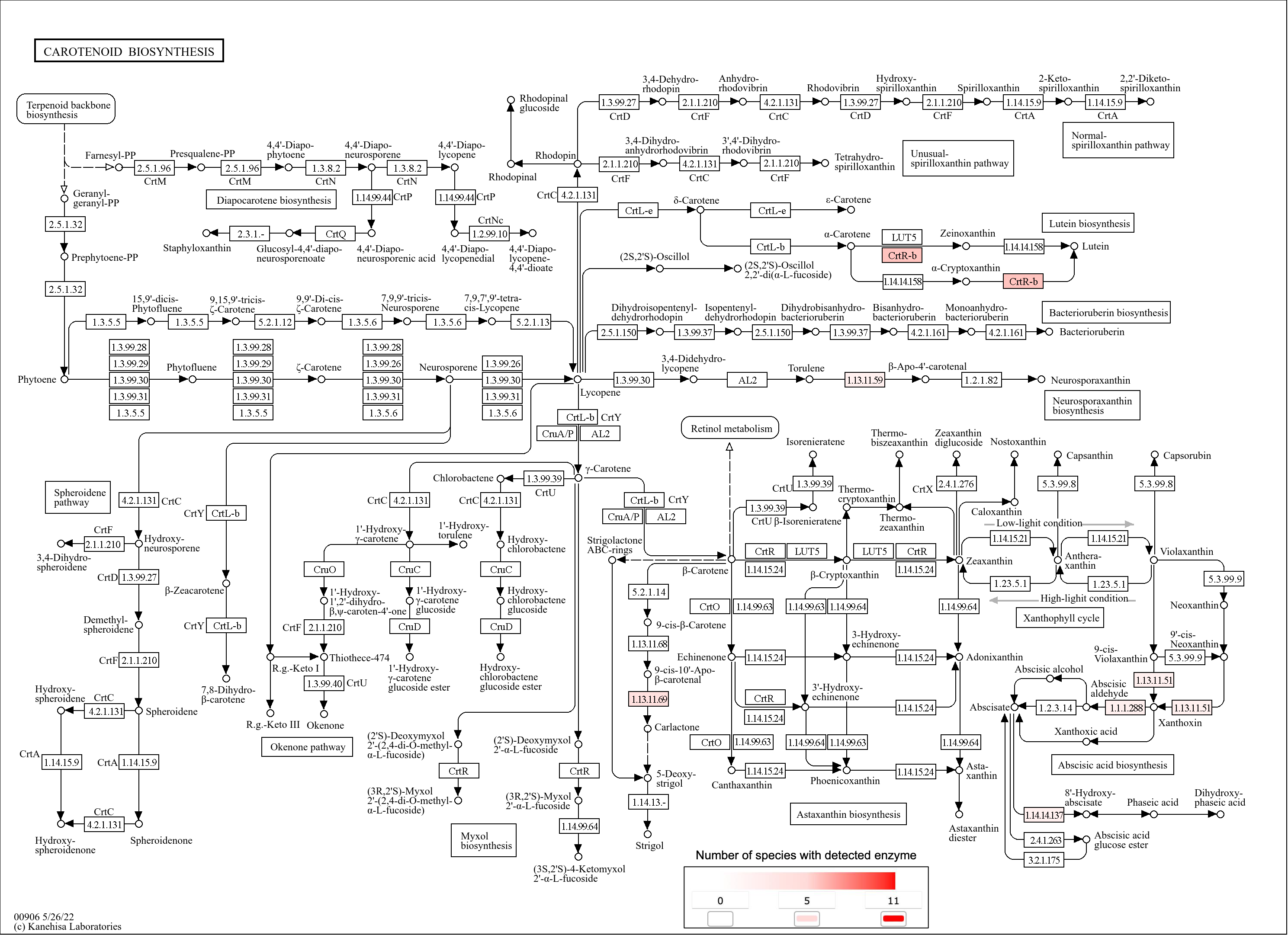

Supplement: Supplementary file 1 [file ijms-25-13172-s001.zip › Supplementary figure S20_map00906@2x_20240919_134238.png]

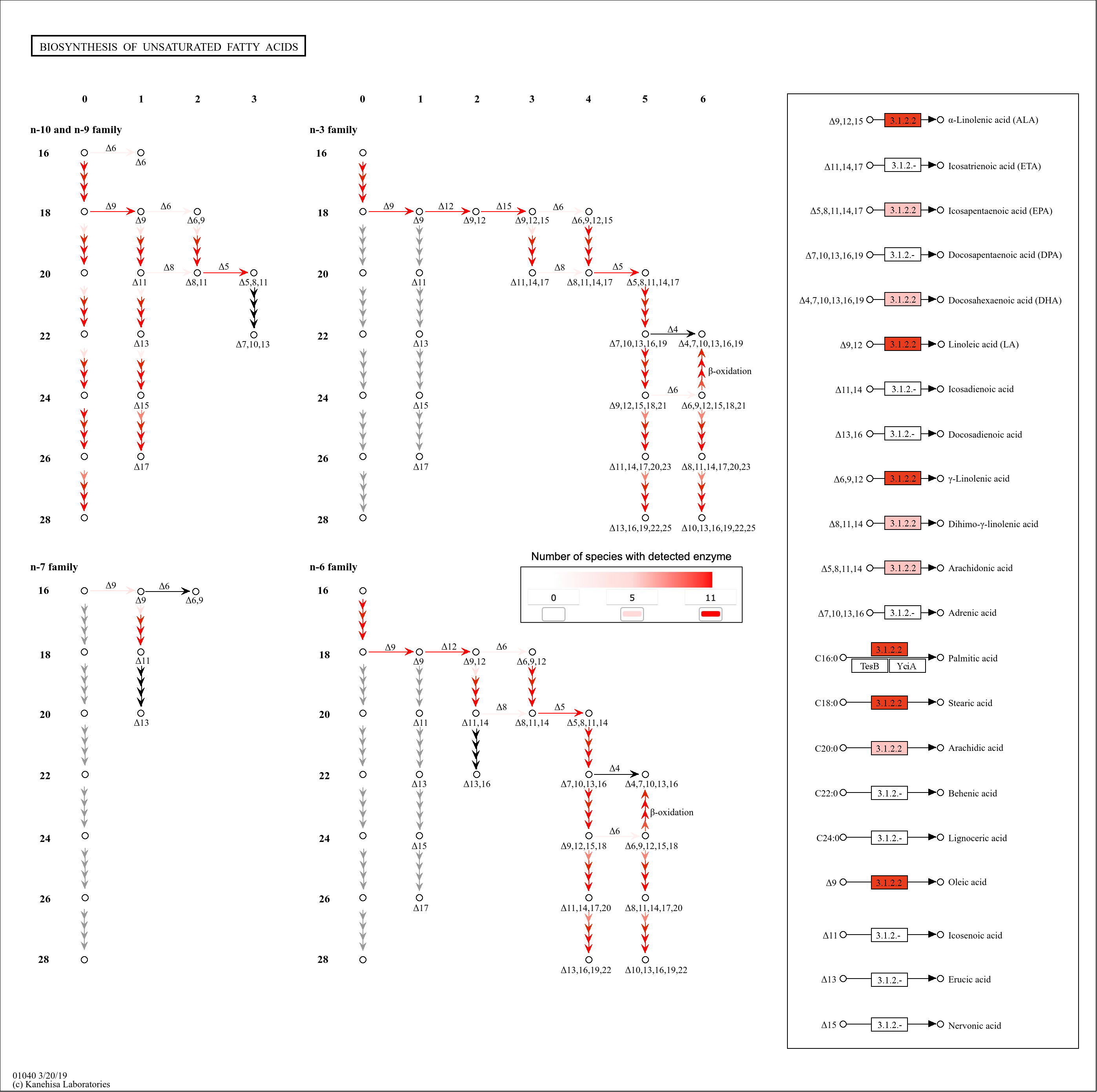

Supplement: Supplementary file 1 [file ijms-25-13172-s001.zip › Supplementary figure S21_map01040@2x_20240923_165436.png]

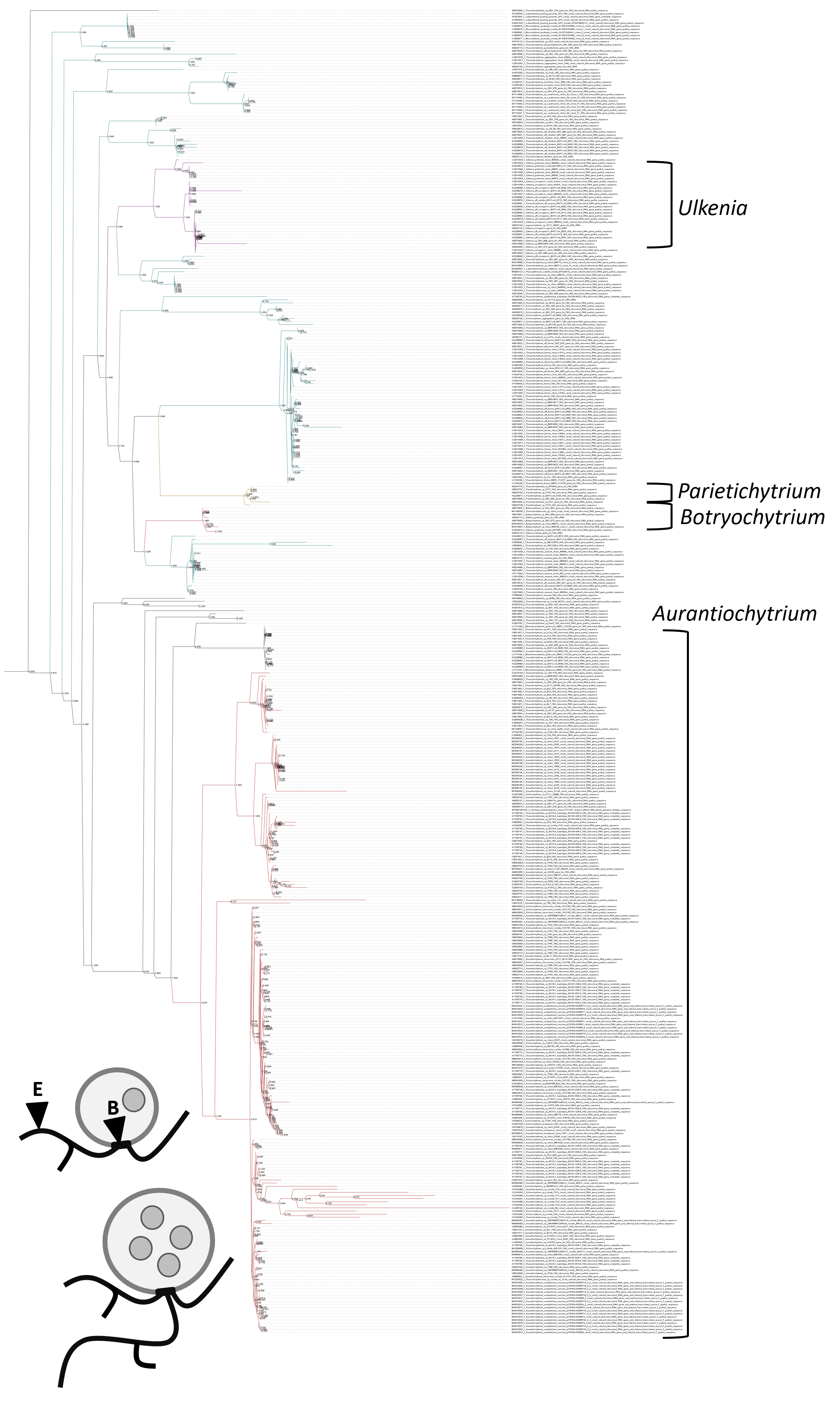

Supplement: Supplementary file 1 [file ijms-25-13172-s001.zip › Supplementary figure S3.tif]

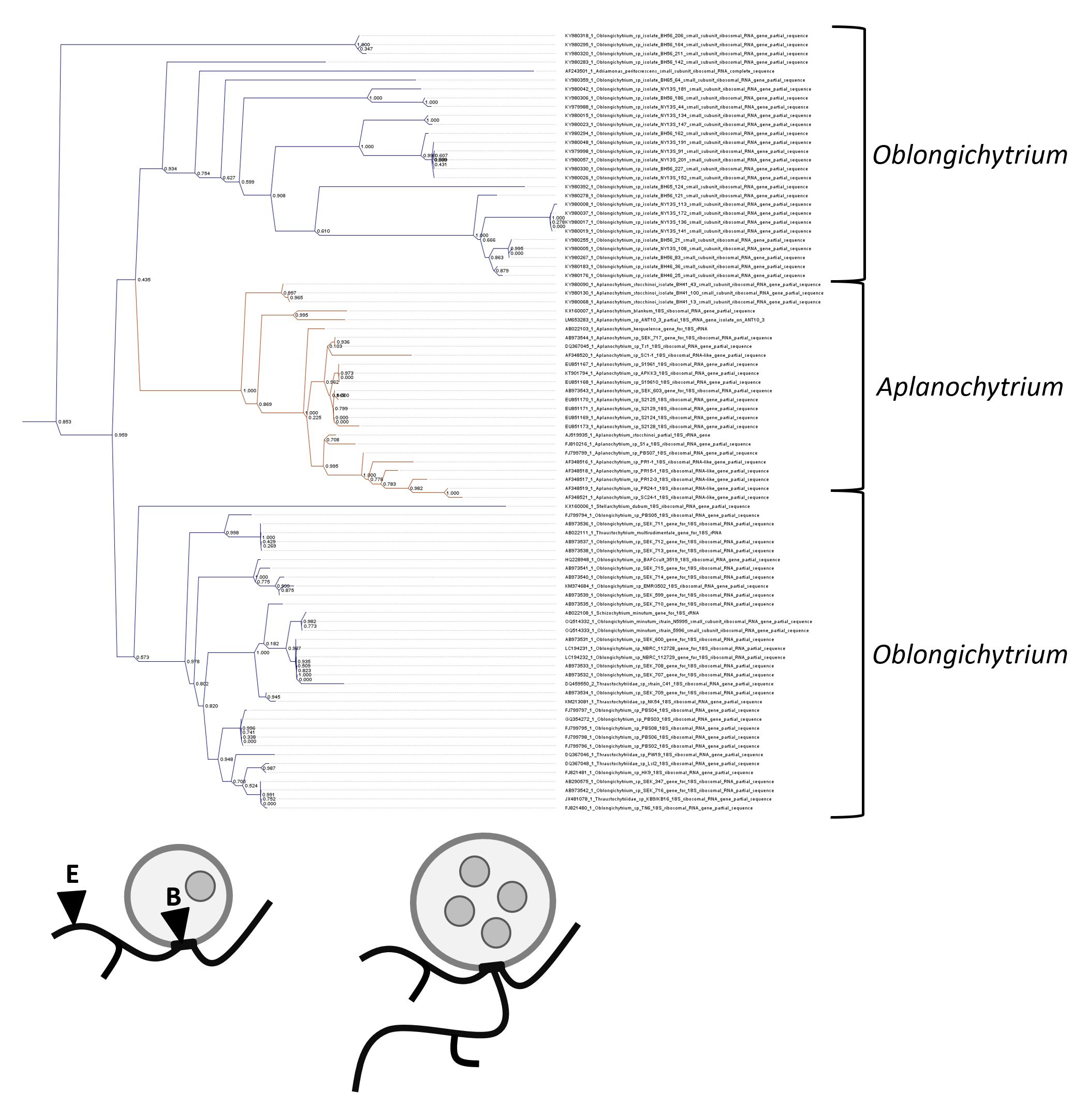

Supplement: Supplementary file 1 [file ijms-25-13172-s001.zip › Supplementary figure S4.tif]

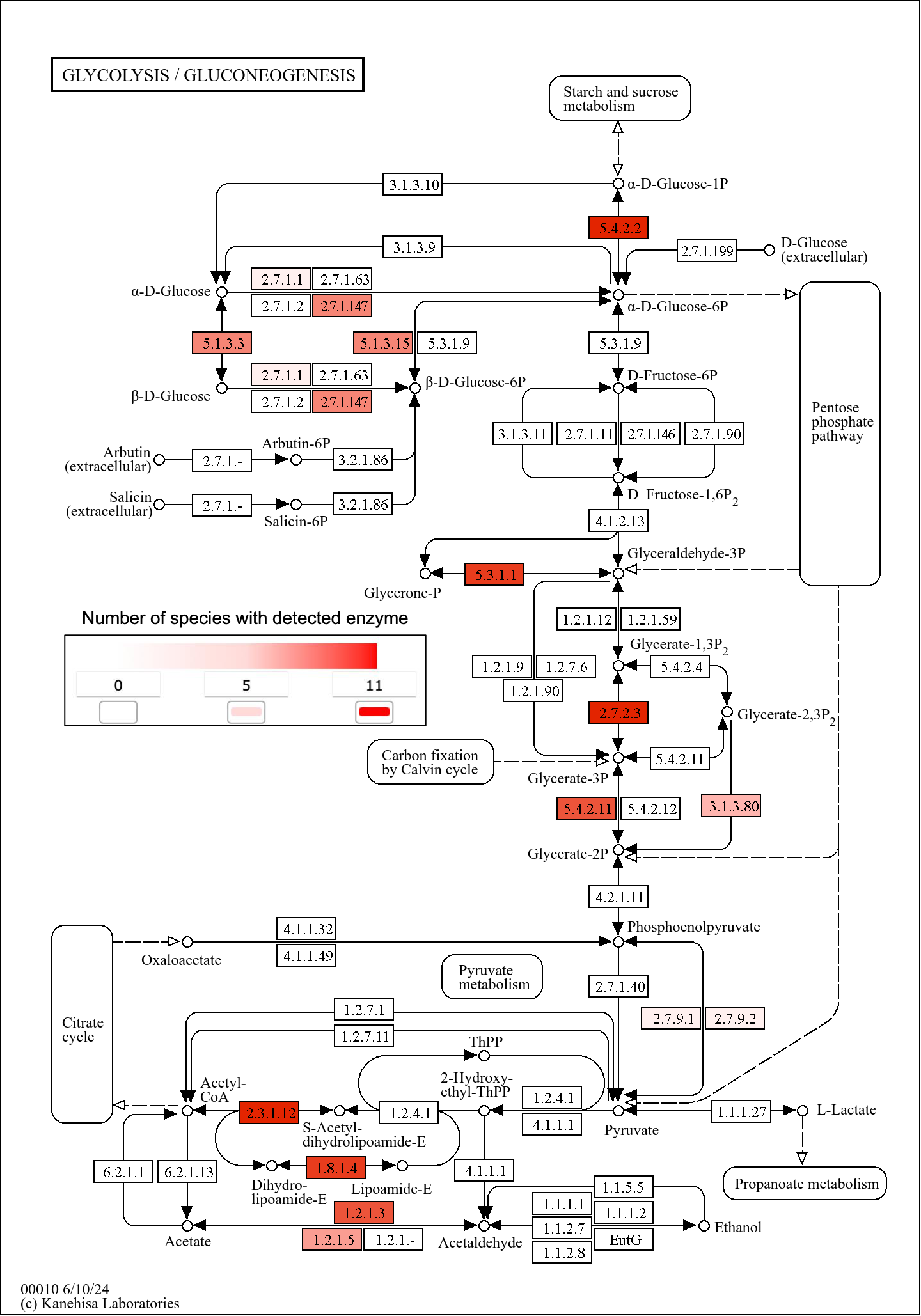

Supplement: Supplementary file 1 [file ijms-25-13172-s001.zip › Supplementary figure S5_map00010@2x_20240919_134050.png]

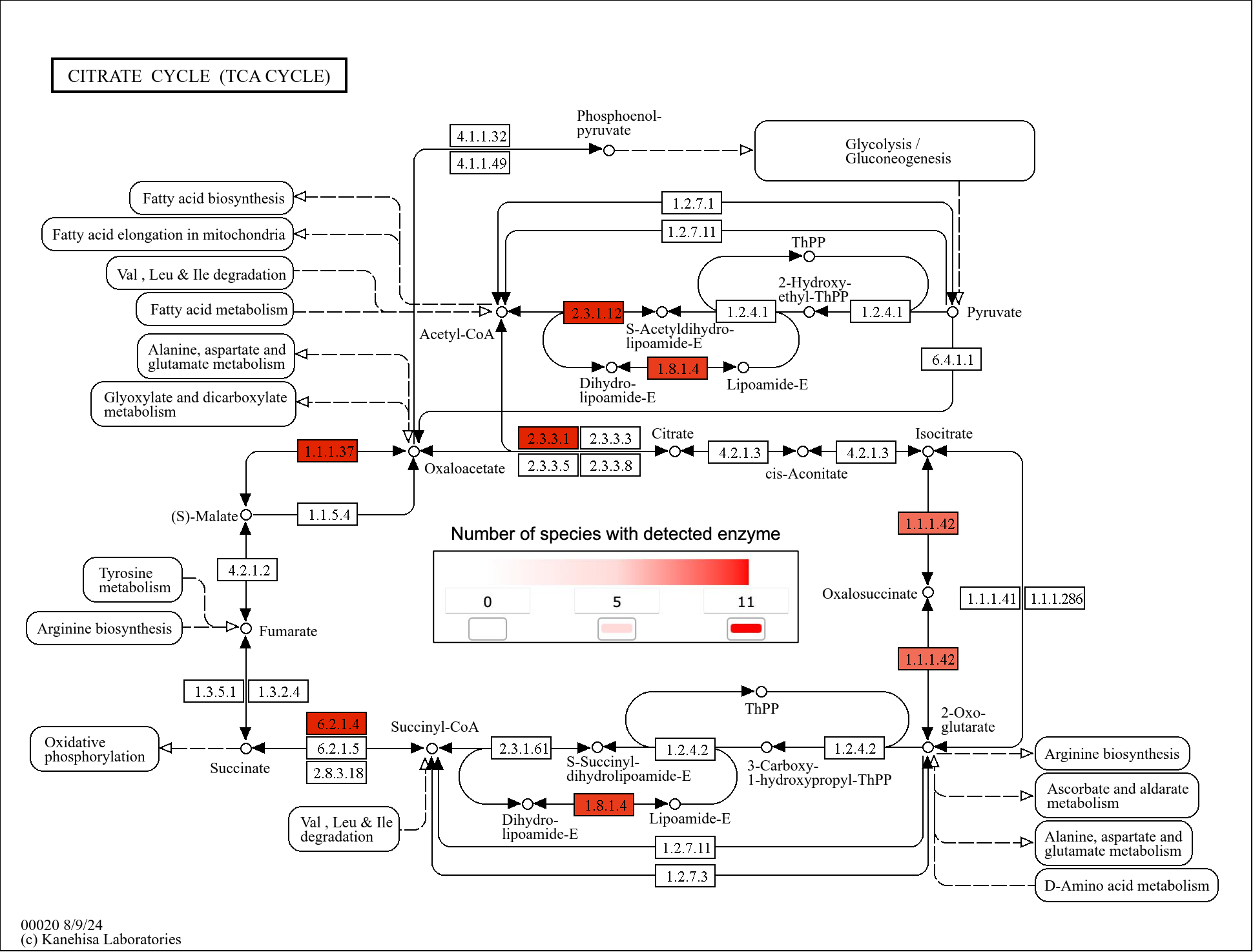

Supplement: Supplementary file 1 [file ijms-25-13172-s001.zip › Supplementary figure S6_map00020@2x_20240919_141926.png]

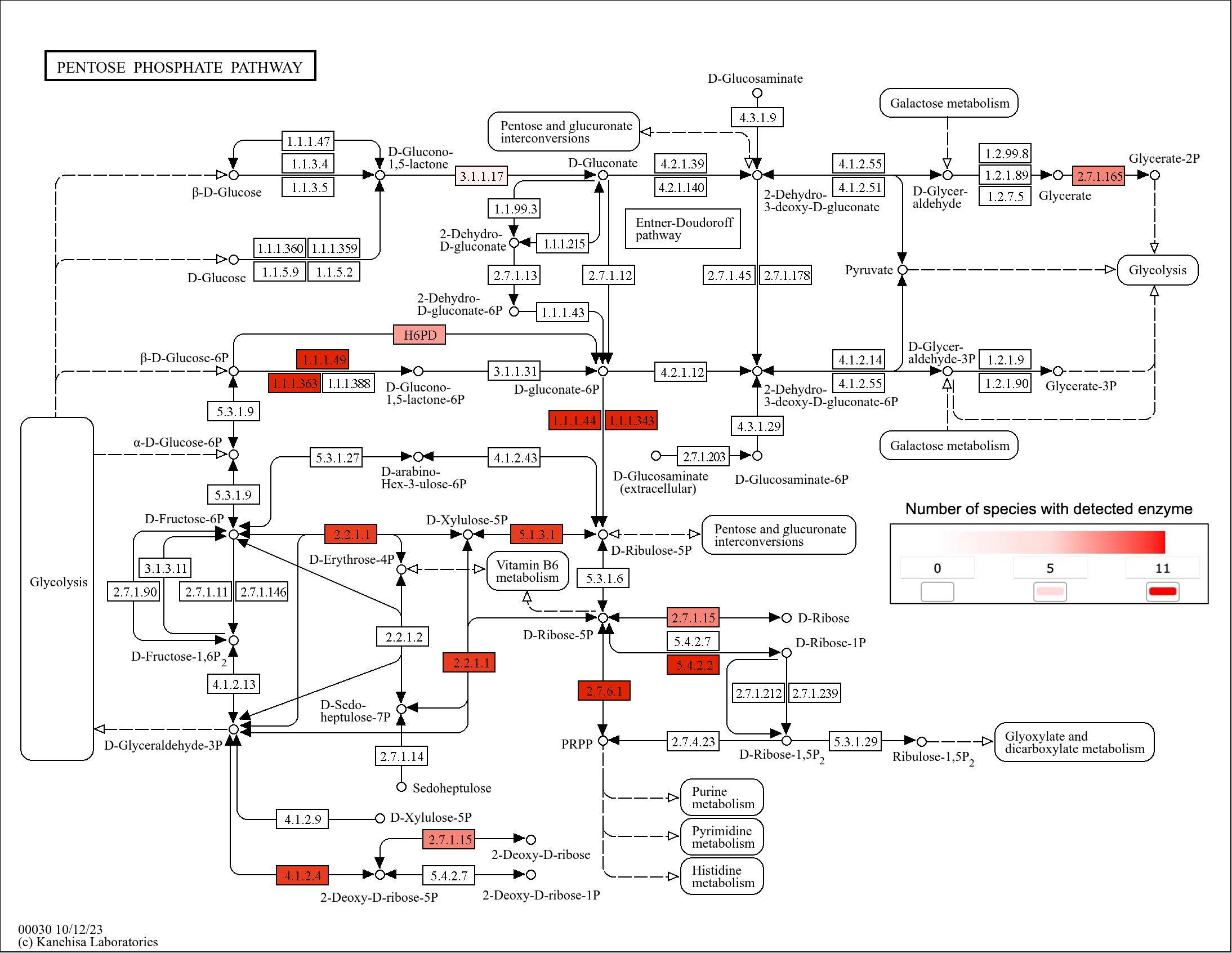

Supplement: Supplementary file 1 [file ijms-25-13172-s001.zip › Supplementary figure S7_map00030@2x_20240919_142043.png]

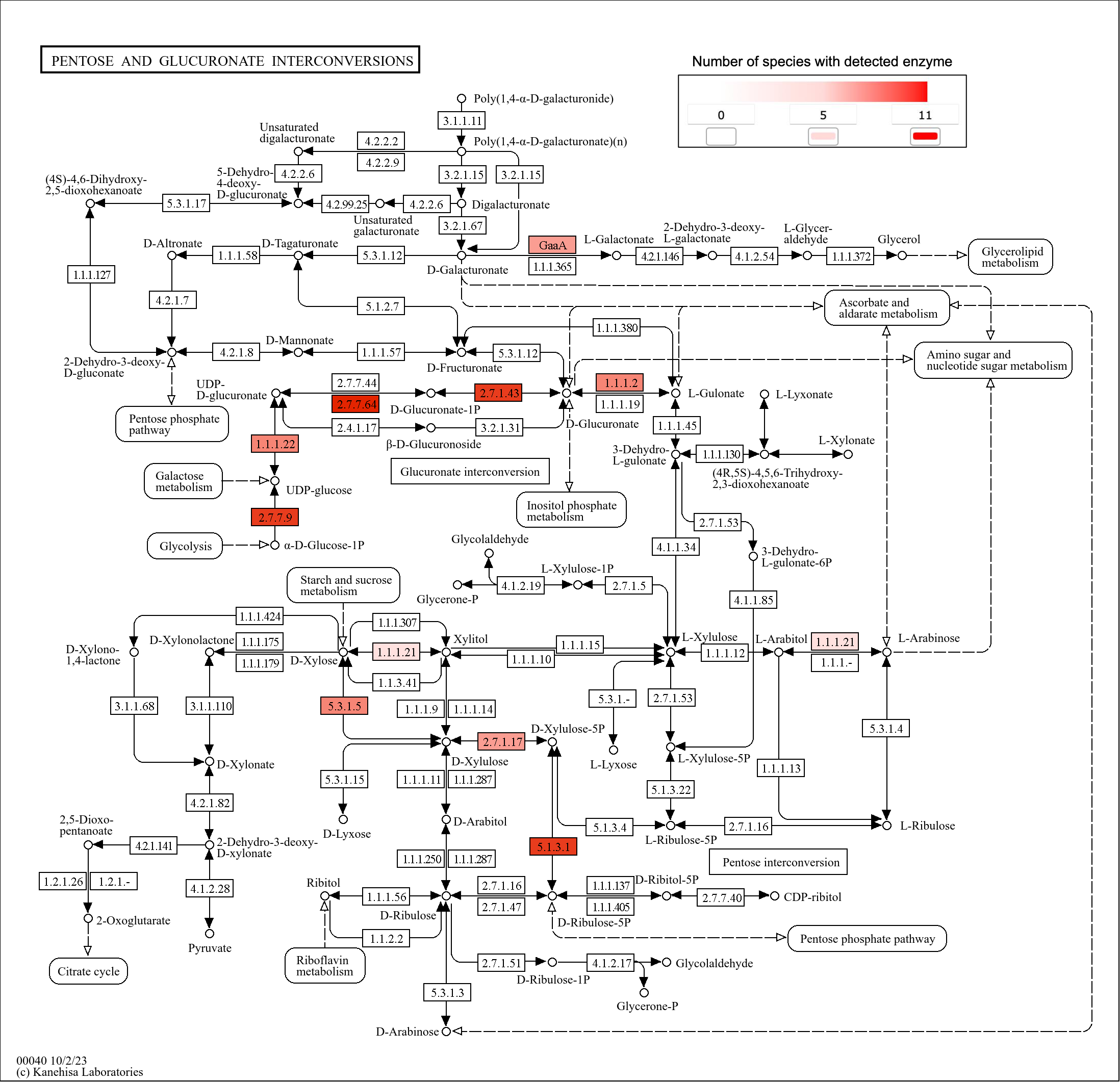

Supplement: Supplementary file 1 [file ijms-25-13172-s001.zip › Supplementary figure S8_map00040@2x_20240919_142224.png]

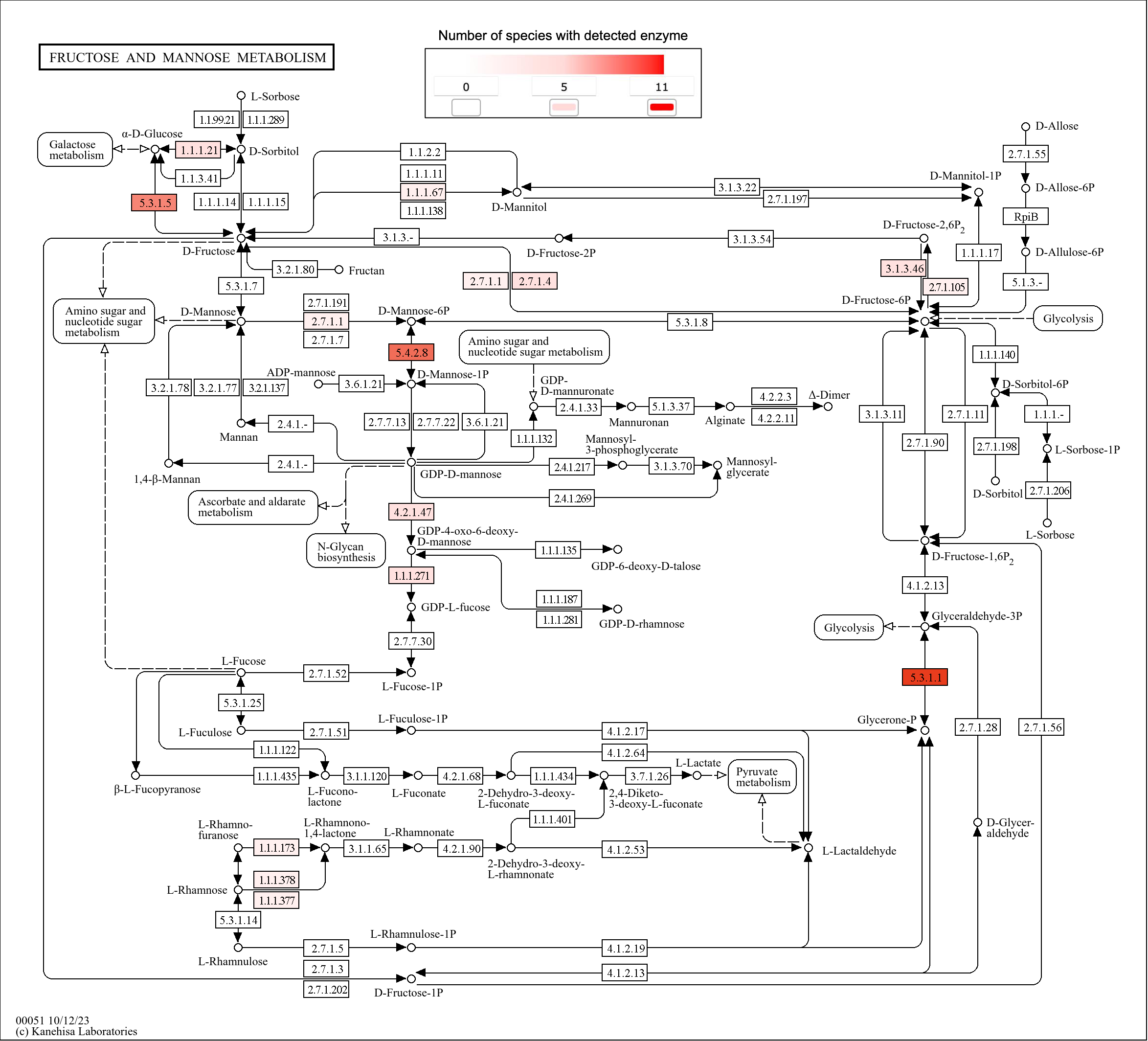

Supplement: Supplementary file 1 [file ijms-25-13172-s001.zip › Supplementary figure S9_map00051@2x_20240919_142333.png]
